# POLE3 is a repressor of unintegrated HIV-1 DNA required for efficient virus integration and escape from innate immune sensing

**DOI:** 10.1126/sciadv.adh3642

**Published:** 2023-11-03

**Authors:** Suzie Thenin-Houssier, Shinichi Machida, Cyprien Jahan, Lucie Bonnet-Madin, Scarlette Abbou, Heng-Chang Chen, Robel Tesfaye, Olivier Cuvier, Monsef Benkirane

**Affiliations:** ^1^ Institut de Génétique Humaine. Laboratoire de Virologie Moléculaire, CNRS Université de Montpellier. Montpellier. France.; ^2^ Department of Structural Virology, National Center for Global Health and Medicine, 1-21-1 Toyama, Shinjuku-ku, Tokyo 162-8655, Japan.; ^3^ Laboratory of Chromatin Dynamics, Centre de Biologie Intégrative (CBI), MCD Unit (UMR5077), Université de Toulouse, CNRS, UPS, 31062 Toulouse, France.

## Abstract

Unintegrated retroviral DNA is transcriptionally silenced by host chromatin silencing factors. Here, we used the proteomics of isolated chromatin segments method to reveal viral and host factors associated with unintegrated HIV-1DNA involved in its silencing. By gene silencing using siRNAs, 46 factors were identified as potential repressors of unintegrated HIV-1DNA. Knockdown and knockout experiments revealed POLE3 as a transcriptional repressor of unintegrated HIV-1DNA. POLE3 maintains unintegrated HIV-1DNA in a repressive chromatin state, preventing RNAPII recruitment to the viral promoter. POLE3 and the recently identified host factors mediating unintegrated HIV-1 DNA silencing, CAF1 and SMC5/SMC6/SLF2, show specificity toward different forms of unintegrated HIV-1DNA. Loss of POLE3 impaired HIV-1 replication, suggesting that repression of unintegrated HIV-1DNA is important for optimal viral replication. POLE3 depletion reduces the integration efficiency of HIV-1. POLE3, by maintaining a repressive chromatin structure of unintegrated HIV-1DNA, ensures HIV-1 escape from innate immune sensing in primary CD4^+^ T cells.

## Teaser

The repressive chromatin structure of unintegrated HIV-1 DNA ensures efficient integration and escape from innate immunity.

## INTRODUCTION

Early in the retroviral life cycle, upon fusion of the viral and cellular membranes and release of the viral core into the cytoplasm, the viral RNA genome is reverse-transcribed into double-stranded linear viral DNA (dslvDNA). Subsequently, this dslvDNA serves as a template for either the host DNA repair machinery to generate circular viral DNA (vDNA) as 2-LTR (long terminal repeat) and 1-LTR circles or for viral integrase (IN) and its cofactors to catalyze its integration into the host genome ensuring viral persistence (*1*, *2*). Newly synthesized unintegrated viral DNAs (uvDNAs) are remarkable in several ways: They are unique in that chromatin has to be configured completely de novo and they are subject to potent transcriptional silencing, in contrast to integrated HIV-1 DNA, which exhibits robust expression (*3*–*5*). This transcriptional silencing is imposed by the establishment of a repressive chromatin structure within unintegrated HIV-1 DNA (uHIV-1 DNA). Loading of HIV-1 DNA with core histones and the repressive linker histone H1 occurs after its complete synthesis and before its integration into the host genome (*3*, *4*). Analysis of the uHIV-1 DNA epigenome revealed the presence of histone repressive marks such as histone 3 lysine 9 trimethylation (H3K9me3) and the absence of H3K4me3, a marker of active promoters, and histone H3 acetylation (H3ac), a marker of active transcription. Determination of nucleosome positioning along the unintegrated and integrated viral genome revealed major differences in nucleosome density and position within the viral LTR. In addition to the well-known nucleosomes Nuc0, Nuc1, and Nuc2 loaded on integrated HIV-1 DNA, a nucleosome covering the deoxyribonuclease (DNase)–hypersensitive site (NucDHS) is present within uHIV-1 DNA. The presence of NucDHS within the promoter proximal region at the LTR interferes with RNA polymerase II recruitment (*3*). Transcriptional activation of HIV-1 DNA is accompanied by NucDHS eviction; loss of repressive and gain of active histone marks such as H3K9me3 and H3Ac, respectively; and recruitment of RNAPII to the viral LTR (*3*). Identifying the host factors involved in establishing and maintaining uHIV-1 DNA in a repressive chromatin state leading to its transcriptional silencing is key to understanding its function during viral replication and its role in viral escape from innate immune sensing. Here, we applied the proteomics of isolated chromatin segments (PICh) method (*6*) to reveal the proteome of linear uHIV-1 DNA. The PICh is a powerful and unbiased method to analyze the composition of chosen chromatin segments, providing a comprehensive knowledge about the steady-state protein composition of the target locus. This method is based on the affinity capture of endogenous chromatin segments by hybridization with specific nucleic acid probes and the identification of the protein associated by mass spectrometry. Using this method, we identified the DNA polymerase epsilon subunit 3 (POLE3) as transcriptional repressor of uHIV-1 DNA that functions by maintaining the viral genome in a chromatin-repressive state. POLE3 is a non-essential subunit of the leading strand polymerase epsilon (POLE) holoenzyme (POLE3/POLE4), a histone H3-H4 chaperone that maintains chromatin integrity during DNA replication (*7*) and is found associated with the chromatin remodeling complex ACF1-SNF2h (*8*). We found selective targeting of uHIV-1 DNA species by POLE3 and the recently identified host factors modulating its transcription (*9*, *10*). Exploring the function of POLE3 in HIV-1 replication revealed key function for viral chromatin structure in its integration into the host genome and escape from innate immune recognition in primary CD4^+^ T cells.

## RESULTS

**Fig. 1.**
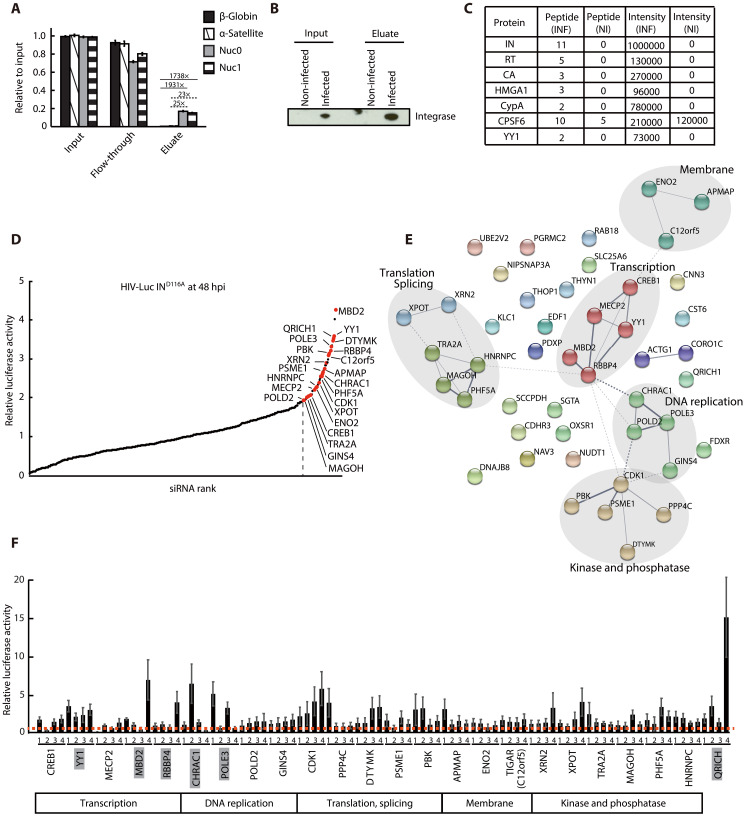
Identification of factors accumulating on uHIV-1 DNA and involved in its silencing. (**A**) qPCR analysis of input, FT, and eluate fractions from PICh isolated from cells infected with HIV-Luc at 9 hpi. Results are presented as quantities relative to input from large-scale experiment with triplicate samples. Enrichment of vDNA over β-globin (full line) and α-satellite (dashed line) is shown. (**B**) Western blot analysis monitoring the presence of the viral IN in the input and eluate fractions. (**C**) Mass spectrometry results for the factors identified by PICh using infected (INF) and noninfected (NI) cells. Number and summed intensity of the peptides matched to viral and host proteins known to play a role in HIV infection or associated with vDNA are presented. (**D**) siRNA screen of host factors silencing uHIV-1 DNA transcription. HeLa cells were transfected with a siRNA library composed of a pool of siRNAs targeting each gene and infected with the HIV-Luc IN^D116A^ virus. Luciferase assays were performed at 48 hpi. The results are presented as luciferase activity relative to nontargeting (NT) siRNA–transfected cells from two independent experiments performed with triplicate samples. Factors to the right of the dotted line were used for interaction network using STRING database. Red indicates factors within functional clusters. (**E**) Protein-protein interaction network of the top-ranked factors identified in (D). Nodes represent proteins. Solid and dotted edges indicate interactions within and between clusters, respectively. Nodes are color-coded based on functional clusters generated by STRING. (**F**) Validation of candidate proteins highlighted in (E). HeLa cells were transfected with four individual siRNAs targeting each gene and infected with HIV-Luc IN^D116A^. Luciferase assay was performed at 48 hpi. Results are presented as luciferase activity relative to that in NT siRNA-transfected cells. Mean ± SD values of two independent experiments with triplicate samples are plotted.

**Fig. 2.**
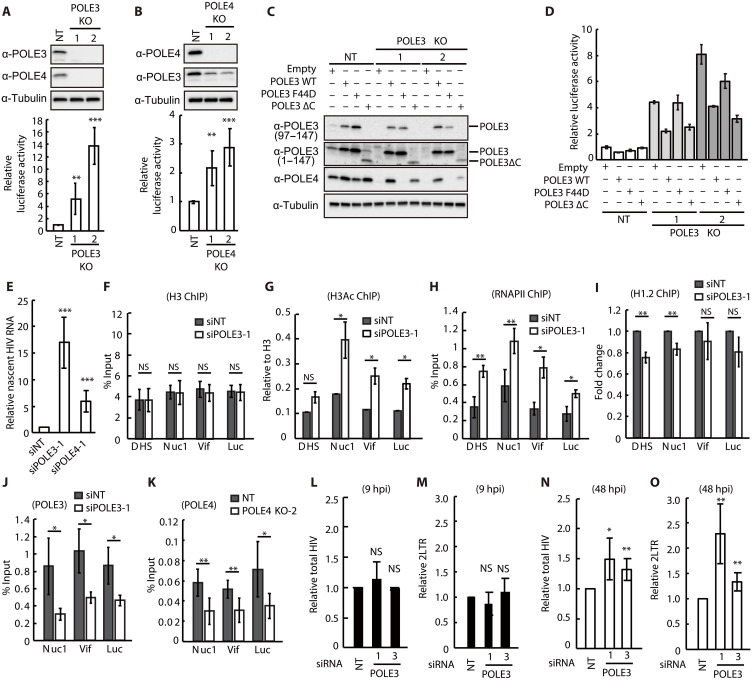
POLE3 and POLE4 are transcriptional repressors of uHIV-1 DNA. (**A** and **B**) Protein expression analysis and luciferase assay results in POLE3 (A) and POLE4 (B) KO cells infected with HIV-Luc IN^D116A^. The results are presented as luciferase activity relative to that in NT single guided RNA (sgRNA) cells. (**C** and **D**) Protein expression analysis (C) and luciferase assay (D) results in POLE3 KO HeLa cells with stable exogenous expression of POLE3 WT, POLE3 F44D, or POLE3 ΔC infected with HIV-Luc IN^D116A^. The results are presented as luciferase activity relative to that in NT sgRNA cells in one representative experiment with triplicate samples. (**E**) Nascent HIV RNA expression from uHIV-1 DNA at 48 hpi in POLE3 and POLE4 KD HeLa cells. The results are presented as nascent HIV RNA relative to NT siRNA. (**F** to **I**) ChIP assay using NT (gray) and POLE3 (white) KD HeLa cells infected with HIV-Luc IN^D116A^ for 48 hours. qPCR analysis was performed using the indicated primers. (**J**) CUT&RUN using NT (gray) and POLE3 (white) KD HeLa cells infected with HIV-Luc IN^D116A^ for 48 hours. qPCR analysis was performed using the indicated primers. (**K**) ChIP assay using NT (gray) and POLE4 (white) KO HeLa cells infected with HIV-Luc IN^D116A^ for 48 hours. qPCR analysis was performed using the indicated primers. (**L** to **O**) Quantification of total HIV-1 DNA (L and N) and 2-LTR circles (M and O) in POLE3 KD HeLa cells infected with HIV-Luc IN^D116A^ at 9 hpi (L and M) and 48 hpi (N and O). The results are presented as quantities relative to those in NT siRNA-transfected cells. Mean ± SD values of at least three independent experiments are plotted. *P < 0.05, **P < 0.01, ***P < 0.001; independent Student’s *t* test. NS, not significant.

To identify host factors that regulate transcription from uHIV-1 DNA, we applied the PICh method (*6*) to preferentially purify linear uHIV-1 DNA from HIV-1–infected SupT1 cells. We designed specific HIV-1 probes covering the 5′-LTR/Gag region of the viral genome. These desthiobiotin oligos are 50 nt long and cover 1.5 kb of the viral genome (table S1). We tested the capture efficiency of these probes in a plasmid pull-down assay using a plasmid expressing the luciferase-encoding gene under the control of the HIV-1 LTR (LTR-luc). After denaturation/hybridization cycle with the specific probes, the plasmid-probes hybrids were captured on streptavidin beads. At this step, the flow-through (FT) was kept. The hybrids were then washed and eluted by competition with biotin. Inputs, FT, and eluates were analyzed on agarose gel. Efficient and specific capture of the LTR-luc fragment was observed using the LTR probes but not with the Gag probes, due to the absence of the Gag sequence in the construct (fig. S1, A to C). Next, we determined the kinetics of the early steps of HIV-1 infection in SupT1 cells. Consistent with previous reports (*3*, *11*, *12*), at 9 hours postinfection (hpi), amounts of 1-LTR circles and 2-LTR circles and integrated forms were low as compared to total HIV-1 DNA, suggesting that the linear uHIV-1 DNA form was predominant (fig. S1D). To monitor the capture efficiency and specificity of the probes in infected cells, we performed PICh experiments using DNA extracted from infected SupT1 cells harvested at 9 hpi. The presence of vDNAs in the input, FT, and eluate was analyzed by quantitative polymerase chain reaction (qPCR) using primers specific for the full-length HIV DNA and 2-LTR circles. The abundance of the housekeeping gene β-globin was quantified as a control. Full-length vDNA was recovered with an efficiency of 24.6% in the eluate and 51.4% in the FT, suggesting that isolation of HIV-1 vDNA was successful, although not complete (fig. S1E). The percentage of recovered 2-LTR circles was 11.65% in the eluate and 63.77% in the FT, highlighting the increased enrichment of linear vDNA (lvDNA) compared to 2-LTR circles in the eluate (fig. S1E). The control β-globin DNA was largely absent in the eluate (2.9%), demonstrating the specificity of the capture assay (fig. S1E). These results demonstrated that the PICh method can be successfully applied for the purification of uHIV-1 DNA with the linear form predominant. In addition, large-scale PICh was performed using chromatin purified from noninfected and VSV-G pseudotyped HIV-luc–infected SupT1 cells at a multiplicity of infection (MOI) of 2 for 9 hours. Cells were crosslinked with 3.6% formaldehyde, and chromatin was prepared by micrococcal nuclease (MNase) digestion. After partial denaturation/hybridization cycles with the HIV-1–specific probes, the chromatin-probe hybrids were captured on streptavidin beads and washed and eluted with biotin. FT were used for qPCR analysis, and inputs and eluates were used for qPCR, Western blot, and mass spectrometry analyses. We measured vDNA capture using LTR primers, and we estimated the relative enrichment of vDNA with respect to β-globin and α-satellite, an abundant tandem repeat sequence in centromeres of human chromosomes. We captured 16.25% of vDNA relative to the input using LTR primers corresponding to the Nuc0 and Nuc1 regions and captured 0.0089 and 0.67% of β-globin and α-satellite DNA, respectively, showing the specific capture of the viral genome (Fig. 1A). We monitored the presence of the viral IN protein by Western blotting and revealed the enrichment of IN in the eluate fraction of infected cells (Fig. 1B), thus validating the PICh strategy. Mass spectrometry analysis identified 459 proteins (table S2) that were enriched in the eluate from infected cells compared to noninfected cells (based on the peptide number and summed intensity), including three viral proteins [IN, capsid (CA), and reverse transcriptase (RT)] and host factors known to bind vDNA or viral proteins (Fig. 1C and table S2) (*2*, *13*–*17*). To investigate the impact of these factors on uHIV-1 DNA silencing, we performed small interfering RNA (siRNA) screens using a custom library of pooled siRNAs targeting the 455 selected candidates. First, the pooled siRNAs were reverse-transfected into HeLa cells, and the toxicity associated with each siRNA was measured. Hit siRNAs resulting in less than 75% viability were excluded (*n* = 13) (table S3). We then investigated the impact of the siRNAs on HIV-1 expression from uHIV-1 DNA using VSV-G pseudotyped HIV-Luc virus bearing a mutation in the catalytic domain of IN (HIV-Luc IN^D116A^). Of the 442 siRNAs tested, 46 resulted in an increase of at least 1.93-fold in the expression of luciferase from uHIV-1 DNA (Fig. 1D and table S4). Notably, factors regulating the expression of uHIV-1 DNA in trans or acting transiently cannot be recovered by the PICh method. Thus, to further investigate candidates involved in the repression of uHIV-1 DNA, we selected 24 candidates based on their biological process (gene ontology), protein-protein interactions, pertinence, and novelty. The selected candidates have been described to be involved in chromatin binding, transcription factor activity, DNA-directed DNA polymerase activity, and RNA binding (Fig. 1E). We individualized these siRNAs and transfected them into HeLa cells infected with HIV-Luc IN^D116A^. Cells transfected with the siRNAs targeting YY1, MBD2, RBBP4, CHRAC1, POLE3, and QRICH1 displayed the highest expression of uHIV-1 IN^D116A^ DNA (Fig. 1F and table S5). Clear correlation between knockdown (KD) efficiency and enhanced expression from HIV-Luc IN^D116A^ was observed for POLE3 but not for CHRAC1 and QRICH (fig. S2, A to C). POLE3, together with POLE4, forms the POLE holoenzyme known as histone H3 and H4 chaperone (*7*). POLE3, together with CHRAC1, interacts with the chromatin remodeling complex ACF1-SNF2h, which is involved in chromatin remodeling and nucleosome positioning (*8*). KD of POLE4 but not KD of ACF1 (fig. S2, D and E) increased HIV-Luc IN^D116A^ expression, suggesting that the POLE3/POLE4 complex but not the ACF1-SNF2H complex is involved in uHIV-1 DNA silencing. We then generated HeLa cells with knockout (KO) of POLE3 and POLE4 using CRISPR-Cas9 gene editing (Fig. 2, A and B, and table S6) and measured uHIV-1 DNA expression upon infection with HIV-Luc IN^D116A^ virus. As observed in KD experiments, POLE3 and POLE4 KO HeLa cells exhibited a significant increase in expression from uHIV-1 DNA compared to control cells (5- to 14-fold increase in POLE3 KO cells and 2.2- to 3-fold increase in POLE4 KO cells) (Fig. 2, A and B). POLE4 expression was completely lost in POLE3 KO HeLa cells, while POLE3 expression was reduced but not completely lost in POLE4 KO cells. We then reconstituted POLE3 expression in POLE3 KO cells with exogenous POLE3; the POLE3 F44D mutant, which cannot interact with POLE4; or the POLE3 ΔC mutant, which lacks histone H3 and H4 chaperone activity (*7*). Reconstitution of POLE3 expression in POLE3 KO cells resulted in a twofold reduction in expression from HIV-Luc IN^D116A^. Exogenous expression of the POLE3 ΔC mutant reduced expression from HIV-Luc IN^D116A^, but exogenous expression of the POLE3 F44D mutant did not (Fig. 2, C and D). These results suggest that POLE3/POLE4-mediated silencing of uHIV-1 DNA is independent of its binding to histones H3 and H4. The repressive activity of POLE3 toward uHIV-1 DNA was also observed in the SupT1 cell line and in hTERT-immortalized RPE-1 human retinal pigment epithelial cells lacking POLE3 (fig. S2, F and G) (*18*), suggesting that this effect is not cell type specific.

Next, to determine whether POLE3 and POLE4 act as transcriptional silencers of uHIV-1 DNA, we analyzed HIV-1 LTR activity in response to KD of POLE3 or POLE4 by quantifying the nascent RNA transcripts (Fig. 2E and fig. S3A). As shown in Fig. 2E, compared to transfection with the nontargeting control siRNA (siNT), KD of POLE3 and POLE4 resulted in increases of 17- and 6-fold, respectively, in transcription from the HIV-Luc IN^D116A^ virus. Transcriptional silencing of uHIV-1 DNA is imposed by interference with RNAPII loading by repressive chromatin modifiers, deposition of active histone marks, and recruitment of repressive H1 linker histones (*3*, *4*). Thus, chromatin prepared from control and POLE3 KD HeLa cells infected with the HIV-Luc IN^D116A^ virus for 48 hours was subjected to chromatin immunoprecipitation (ChIP). We assessed the presence of HIV-1 DNA and control genomic loci in the immunoprecipitated chromatin by qPCR using specific primers. The specificity of the anti-H3Ac, anti-RNAPII, and anti-H1.2 antibodies was confirmed using positive and negative controls corresponding to well-characterized genomic loci [positive control: the glyceraldehyde phosphate dehydrogenase (GAPDH) promoter, which is enriched with H3Ac and RNAPII and not bound by H1.2; negative control: B13 and α-satellite, which are not bound by H3Ac and RNAPII but are bound by H1.2] (fig. S3, B to E). While POLE3 KD had no effect on H3 loading (Fig. 2F), it did increase by 2.1-fold the level of the active histone mark H3Ac (Fig. 2G) and the recruitment of RNAPII (Fig. 2H) along the HIV-1 genome. A small but statistically significant reduction in H1.2 loading at HIV-1 regulatory regions was observed in POLE3 KD cells compared to control siNT-transfected cells (Fig. 2I). Next, the accumulation of POLE3 and POLE4 on uHIV-1 DNA was investigated by cleavage under targets and release using nuclease (CUT&RUN) and ChIP, respectively. As shown in Fig. 2 (J and K), vDNA sequences were recovered in POLE3 and POLE4 IPs using chromatin prepared from siNT control cells, suggesting that POLE3 and POLE4 are loaded onto uHIV-1 DNA. The presence of vDNA was reduced in POLE3 and POLE4 IPs when using chromatin prepared from POLE3 KD or POLE4 KO cells, witnessing the specificity of the used antibodies. Together, our results showed that POLE3 and POLE4 directly target and transcriptionally silence uHIV-1 DNA by imposing a repressive chromatin structure.

To investigate whether POLE3 affects HIV-1 DNA synthesis, HeLa cells transfected with NT or POLE3 siRNA were infected with HIV-Luc IN^D116A^ virus, and the synthesis of total HIV DNA and the formation of 2-LTR circles were analyzed at 9 and 48 hpi. Total HIV-1 DNA and 2-LTR circle formation were not affected at 9 hpi (Fig. 2, L and M), while significant increase in both was observed at 48 hpi in POLE3 KD cells compared to siNT control cells (Fig. 2, N and O), suggesting that POLE3 may negatively affect HIV-1 DNA stability and/or 2-LTR circle formation.

**Fig. 3.**
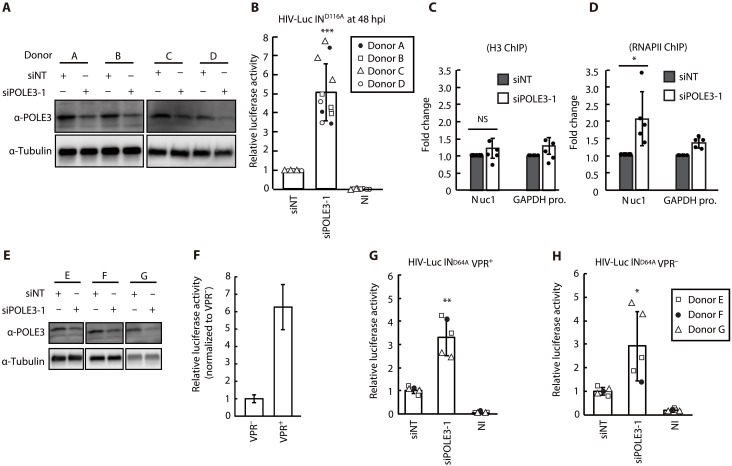
POLE3 silences uHIV-1 DNA transcription in primary CD4^+^ T cells. (**A** and **B**) PHA–IL-2–activated primary CD4^+^ T cells from four healthy donors were electroporated with NT and POLE3 siRNAs and then infected with VSV-G pseudotyped HIV-Luc IN^D116A^. POLE3 expression in whole-cell extracts on the day of infection was analyzed by immunoblotting using an anti-POLE3 antibody (A). Luciferase assays were performed at 48 hpi (B). The results are presented as luciferase activity relative to that in NT siRNA-transfected cells; the mean ± SD values of triplicate samples from four healthy donors are plotted. (**C** and **D**) ChIP assays were performed at 48 hpi using anti-H3 (C) and anti-RNAPII antibodies (D). qPCR analysis was performed using specific primers for Nuc1 and the GAPDH promoter (control genomic region). The results are presented as the fold change between NT siRNA-transfected (gray) and POLE3 siRNA-transfected (white) cells, and the mean ± SD values of five independent experiments are plotted. (**E** to **H**) POLE3 silences uHIV-1 DNA transcription in a VPR-independent manner. PHA–IL-2–activated primary CD4^+^ T cells from three healthy donors were electroporated with NT and POLE3 siRNAs and then infected with VSV-G pseudotyped HIV-Luc IN^D64A^ with (G) or without (H) VPR expression. The expression of POLE3 in whole-cell extracts on the day of infection was analyzed by immunoblotting (E). Luciferase assays were performed at 48 hpi (F to H). The results are presented as luciferase activity relative to that in NT siRNA-transfected cells; the mean ± SD values of each duplicate from three healthy donors are plotted. *P < 0.05, **P < 0.01, ***P < 0.001; independent Student’s *t* test.

Next, we analyzed POLE3-mediated transcriptional silencing of uHIV-1 DNA in primary CD4^+^ T cells. CD4^+^ T cells isolated from four healthy donors were activated with phytohemagglutinin/interleukin-2 (PHA–IL-2), transfected with NT or POLE3 siRNA, and subsequently infected with the HIV-Luc IN^D116A^ virus. Partial but consistent POLE3 KD was observed (Fig. 3A). An average increase of fivefold in luciferase activity was observed in POLE3 KD primary CD4^+^ T cells compared to the corresponding siNT control cells (Fig. 3B). Consistent with the data shown in Fig. 2 (F and H), the results of the ChIP assay using anti-H3 and anti-RNAPII antibodies showed that POLE3 KD in primary CD4 T cells did not affect H3 loading and significantly increased (2.1-fold) RNAPII recruitment at the Nuc1 region of the HIV-1 genome (Fig. 3, C and D). Previous studies have shown that the HIV-1 accessory protein R (VPR) relieves uHIV-1 DNA silencing (*19*, *20*) by antagonizing the activity of SMC5/SMC6/SLF2 complex (*9*). Therefore, we assessed the interdependence of POLE3 to VPR in regulating transcription from uHIV-1 DNA in primary CD4^+^ T cells. We used cells isolated from three healthy donors that we transfected with NT or POLE3 siRNA (Fig. 3A) and infected with VPR^+^ or VPR^−^ IN mutant viruses (HIV-Luc IN^D64A^ VPR +/−). While VPR increased transcription from uHIV-1 DNA in primary CD4^+^ T cells (Fig. 3F), POLE3 KD increased luciferase activity compared to that in control cells infected with either the VPR^+^ or VPR^−^ virus (Fig. 3, G and H). Together, our data revealed that POLE3 is a transcriptional repressor of uHIV-1 DNA in different cell types including primary CD4^+^ T cells and that the effect of POLE3 is independent of VPR.

**Fig. 4.**
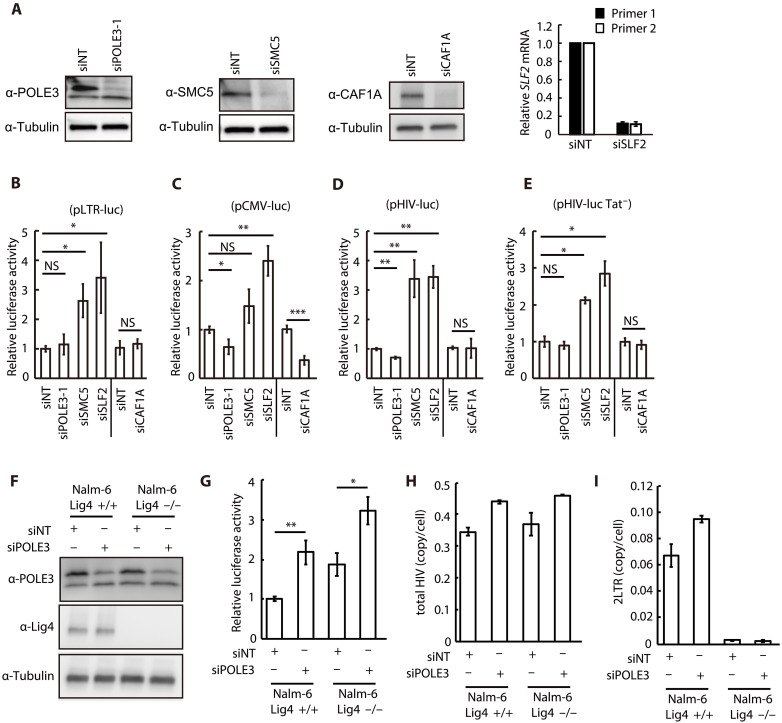
POLE3 does not affect expression from plasmid DNA. (**A** to **E**) Effects of POLE3, SMC5, SLF2, and CAF1 KD on expression from plasmid DNA. POLE3, SMC5, and CAF1A expression from whole-cell extracts on the day of plasmid transfection was analyzed by immunoblotting. The amount of SLF2 mRNA transcript on the day of plasmid transfection was measured by RT-qPCR using two different primer sets, as no antibody was available for Western blot analysis. A luciferase assay using POLE3, SMC5, SLF2, and CAF1A KD HeLa cells transfected with pLTR-Luc (B), pCMV-Luc (C), pHIV-Luc (D), and pHIV-Luc Tat^−^ (E) was performed 24 hours posttransfection. The results are presented as luciferase activity relative to that in NT siRNA-transfected cells, and the mean ± SD values of four independent experiments are plotted. (**F** to **I**) Silencing of uHIV-1 DNA by POLE3 in the absence of the 2-LTR circle form. Expression of POLE3 protein in Nalm-6 (*Lig4* +/+ or −/−) cells electroporated with POLE3 siRNA (F). Luciferase assays were performed in Nalm-6 (*Lig4* +/+ or −/−) POLE3 KD cells infected with VSV-G pseudotyped HIV-Luc IN^D116A^ at 48 hpi (G). The results are presented as luciferase activity relative to that in Nalm-6 (*Lig4* +/+) cells electroporated with NT siRNA, and the mean ± SD values of four independent experiments are plotted. Quantification of total HIV DNA (H) and 2-LTR circles (I) in Nalm-6 (*Lig4* +/+ or −/−) POLE3 KD cells infected with HIV-Luc IN^D116A^ at 48 hpi. The results are presented as the copy number per cell, and the mean ± SD values of two independent experiments are plotted. *P < 0.05, **P < 0.01, ***P < 0.001; independent Student’s *t* test.

To assess the specificity of POLE3-dependent silencing, we first measured the impact of POLE3 depletion on the expression of integrated HIV-1 DNA. We used HeLa cells containing integrated HIV-1 expressing luciferase and lacking the *env* and *tat* genes (HeLa-pl376). POLE3 KD in these cells had no effect on expression from integrated HIV-1 DNA (fig. S4A). We also assessed the impact of POLE3 depletion on unintegrated murine leukemia virus (uMLV) DNA (*21*) and found that POLE3 depletion had a marginal effect on MLV carrying an invalidating IN mutation (MLV-Luc IN^D184A^) compared to HIV-1 IN^D116A^ (fig. S4B). uHIV-1 DNA exists in two forms: circular DNA containing either one copy or two copies of the LTR (1-LTRc or 2-LTRc) and linear DNA, which is the substrate for integration into host DNA. Therefore, we questioned the specificity of POLE3 and the recently identified host factor repressors of uHIV-1 DNA (*9*, *10*) toward these forms of uHIV-1 DNA. For this purpose, we measured expression from HIV LTR-driven or human cytomegalovirus (CMV) promoter-driven luciferase-reporter plasmid DNA (pLTR-luc and pCMV-luc, respectively), a wild-type (WT) HIV molecular clone (pHIV-luc), and the Tat minus variant of this clone (pHIV-luc Tat^−^) in cells with depletion of POLE3, SMC5, SLF2, and CAF1 (Fig. 4A). KD of SMC5 and SLF2 resulted in increased expression from all the plasmid DNAs, including pCMV-luc; however, KD of POLE3 and CAF1A had no effect on expression from any plasmid DNA compared to that in siNT control cells (Fig. 4, B to E). Next, we exploited the well characterized Nalm6 cell model, in which the absence of Ligase 4 (Lig4) results in the loss of HIV-1 2-LTR circle formation (Fig. 4, F and I) (*22*, *23*), to assess the contribution of these host factors to POLE3 activity toward uHIV-1 DNA. POLE3 KD increased expression from uHIV-1 DNA in Lig4 KO Nalm-6 cells (Fig. 4G), whereas the formation of 2-LTR circles was markedly reduced (Fig. 4I). Notably, the results for SMC5 and CAF1A were inconclusive, since we were unable to achieve their efficient KD in the Nalm6 model. Together, these results showed that POLE3 and CAF1 specifically silence reverse-transcribed uHIV-1 DNA.

**Fig. 5.**
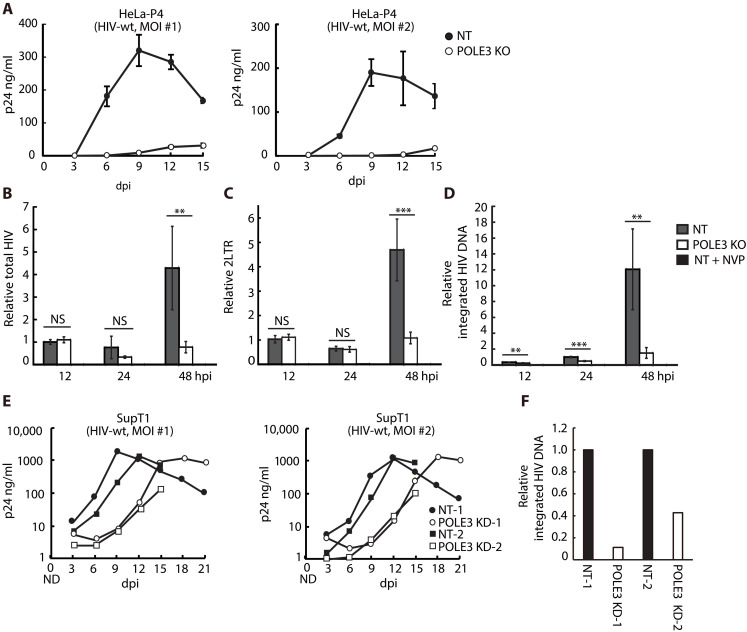
Depletion of POLE3 markedly impairs HIV-1 replication kinetics. (**A**) Effect of POLE3 depletion on HIV-1 replication in HeLa-P4 cells. POLE3 KO HeLa-P4 (black circle) and control (white circle) cells were infected with replication-competent HIV-1 (NL4.3) at two different MOIs. Supernatants were collected every 3 days. HIV-1 spreading was quantified by measurement of the p24 concentration in the culture medium. (**B** to **D**) Effect of POLE3 depletion on HIV-1 integration in HeLa-P4 cells. Quantification of total HIV DNA (B), 2-LTR circles (C), and integrated provirus (D) in HeLa-P4 POLE3 KO cells infected with replicative HIV-1 (NL4.3; MOI = 0.5). The results are presented as quantities relative to those in control cells at 12 hpi (C and D) or 24 hpi (E), and the mean ± SD values of two independent experiments with triplicate samples are plotted. (**E**) Effect of POLE3 depletion on HIV-1 replication in SupT1 cells. POLE3 KD (black circle and square) and control (white circle and square) SupT1 cells were infected with replication-competent HIV-1 (NL4.3) at two different MOIs. Supernatants were collected every 3 days. HIV-1 spreading was quantified by measurement of the p24 concentration in the culture medium. (**F**) Effect of POLE3 KD on HIV-1 integration in SupT1 cells. Integrated HIV DNA was quantified using samples from the HIV-1 spreading assay (MOI 0.05) at 3 days postinfection (dpi). The results are presented as quantities relative to those in NT control cells. **P < 0.01, ***P < 0.001; independent Student’s *t* test. ND, not determined.

The observation that POLE3, unlike SMC5 and SLF2, does not affect expression from either circular plasmid DNA nor 2-LTR circles suggests its repressive activity toward linear uHIV-1 DNA transcription, although we could not exclude a role from the 1-LTR circles. Given the different contributions of the uHIV-1 DNA forms (circular and linear) to the viral life cycle, the specific derepression of uHIV-1 DNA forms may have different impacts on viral replication. Thus, we investigated how the loss of POLE3 may affect viral replication. For this purpose, HeLa CD4 (HeLa-P4) POLE3 KO (fig. S5, A and B, and table S6) and SupT1 POLE3 KD cells (fig. S2F) were infected with WT (HIV-1 (NL4.3) at 2 different MOIs, and viral replication was monitored every 3 days by measuring the p24 viral antigen content in the culture supernatant. Unexpectedly, after infection at both MOIs, HIV-1 replication kinetics were markedly impaired in HeLa-P4 POLE3 KO cells (Fig. 5A) and delayed in SupT1 POLE3 KD cells (Fig. 5E) compared to control cells. How may POLE3 function as factor facilitating HIV-1 replication while it represses transcription from uHIV-1 DNA? We hypothesized that by preventing the loading of the transcriptional machinery, POLE3 may ensure efficient integration of the lvDNA and/or escape from innate immune sensing. Thus, we sought to identify the step of the viral life cycle affected by loss of POLE3. Analyses of vDNA synthesis and integration in HeLa-P4 POLE3 KO cells infected with the NL4.3 virus revealed that the content of total HIV-1 DNA (Fig. 5B) and HIV-1 DNA 2-LTR circles (Fig. 5C) were similar between POLE3 KO and control cells at 12 and 24 hpi, while integration of vDNA into the host genome was reduced by 50% at 24 hpi (Fig. 5D). Thus, the content of total HIV-1 DNA, 2-LTR circles, and integrated vDNA were reduced during the subsequent replication cycle in POLE3 KO cells, as measured at 48 hpi (Fig. 5, B to D). Similarly, decreased vDNA integration was observed in SupT1 POLE3 KD cells (Fig. 5F). Depletion of POLE3 had no effect on the late steps of viral replication, namely, viral release (fig. S6, A to D) and infectivity (fig. S6, E and F). Together the results of these experiments suggested that POLE3 is an HIV-1–facilitating factor that ensures efficient viral integration by preventing transcription from uHIV-1 DNA, although we could not exclude that other mechanism might be involved.

**Fig. 6.**
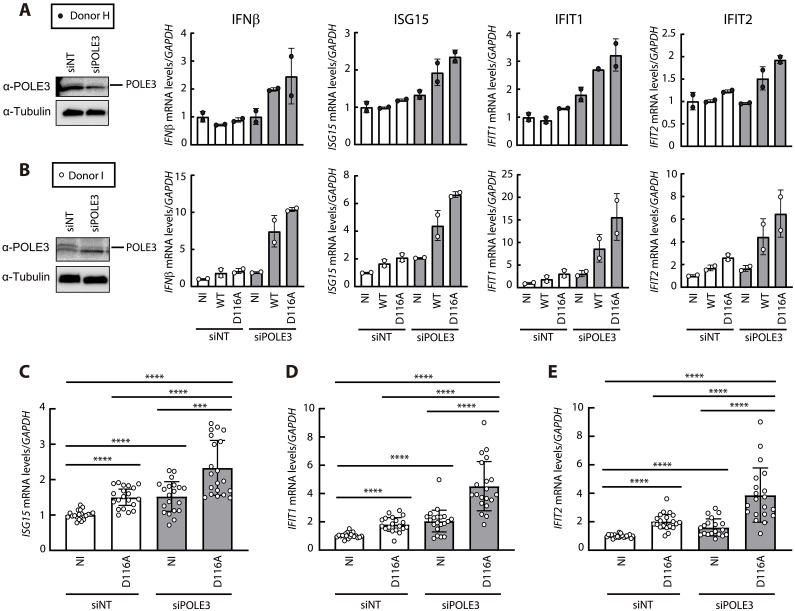
Depletion of POLE3 induces an innate immune response in primary CD4^+^ T cells. (**A** and **B**) PHA–IL-2–activated primary CD4^+^ T cells isolated from two healthy donors were electroporated with NT (white) and POLE3 (gray) siRNAs and then infected with VSV-G pseudotyped HIV-Luc INwt or IN^D116A^ in duplicate. POLE3 expression in whole-cell extracts on the day of infection was analyzed by immunoblotting using an anti-POLE3 antibody. IFNβ, ISG15, IFIT1, and IFIT2 mRNA expression was measured by RT-qPCR at 24 hpi. (**C** to **E**) PHA–IL-2–activated primary CD4^+^ T cells isolated from 10 additional donors were infected with HIV-Luc IN^D116A^. ISG15 (C), IFIT1 (D), and IFIT2 (E) mRNA expression was measured by qPCR at 24 hpi. Duplicate infections for each donor are presented. The results are presented relative to siNT-transfected noninfected cells (NI). *P < 0.05, **P < 0.01, ***P < 0.001, ****P < 0.0001; Mann-Whitney *U* test.

Previous studies have shown that HIV-1 infection fails to induce detectable type I interferon (IFN) expression in activated primary CD4^+^ T cells and in monocyte-derived macrophages (*24*–*27*). To explore the potential function of POLE3 in innate immune sensing of HIV-1, PHA–IL-2–activated CD4^+^ T cells isolated from two healthy donors were transfected with NT or POLE3 siRNA and subsequently infected with the VSV-G pseudotyped HIV-Luc IN^wt^ or IN^D116A^ virus. The expression of IFNβ and IFN-induced genes was quantified by quantitative reverse transcription PCR. As previously shown, HIV-1 infection of primary CD4^+^ T cells failed to induce or very weakly induced (in cells from donor I) type I IFN production and the IFN-responsive genes *ISG15*, *IFIT1*, and *IFIT2* (Fig. 6, A and B). We observed induction of *IFNβ*, *ISG15*, *IFIT1*, and *IFIT2* in POLE3 KD primary CD4^+^ T cells infected with either the HIV-Luc IN^wt^ or HIV-Luc IN^D116A^ virus compared to uninfected control cells (Fig. 6, A and B). Innate immune response induction does not require viral integration, since HIV-1 IN^D116A^ efficiently induced type I IFN production and IFN-responsive genes (Fig. 6, A and B). Although increases were observed in CD4^+^ T cells isolated from both donors, the increases in the levels of HIV-1–induced type I IFN and IFN-responsive genes varied, ranging from 2-fold to 15-fold (Fig. 6, compare A to B). Thus, a similar experiment was performed using CD4^+^ T cells isolated from 10 additional donors (Fig. 6, C to E). HIV-Luc IN^D116A^ infection of siNT control cells resulted in low but significant induction of *ISG15*, *IFIT1*, and *IFIT2* compared to that in noninfected control cells. Similarly, in the absence of infection, POLE3 KD resulted in low but significant induction of the IFN response compared to that in siNT control cells, suggesting a role of POLE3 in regulating the innate immune response. HIV-LucIN^D116A^ infection of POLE3 KD primary CD4^+^ T cells resulted in an increased (2.3- to 4.5-fold) type I IFN response compared to that in noninfected and HIV-1 IN^D116A^-infected control CD4^+^ T cells (Fig. 6, C to E). Together, these experiments identified POLE3 as a host factor used by HIV-1 to escape the innate immune response in primary CD4^+^ T cells.

## DISCUSSION

In this study, proteomic analysis of isolated uHIV-1 DNA revealed POLE3 as a transcriptional silencer of uHIV-1 DNA and a key player in the escape of HIV-1 from innate immune recognition of its unintegrated DNA in primary CD4^+^ T cells. POLE3 and POLE4 are the nonessential subunits of the leading-strand POLE holoenzyme and histone H3-H4 chaperone involved in nucleosome assembly during DNA replication (*7*). A switch of uHIV-1 DNA from a repressive to an active chromatin state with enhanced RNAPII recruitment is observed upon POLE3 KD. The observation that POLE3 KD did not affect H3 loading onto uHIV-1 DNA and that its H3 and H4 histone binding domain (C-terminal domain) is not required for uHIV-1 DNA silencing suggest the existence of a yet-to-be-identified host factor responsible for POLE3 recruitment to the viral genome. Our work suggests a specificity of host silencing factors toward unintegrated retroviral DNA and toward different forms of uHIV-1 DNA. Transcriptional silencing of uMLV DNA and uHIV-1 DNA involves different host factors, with the NP220/HUSH complex targeting MLV (*28*), while POLE3 and CAF1 (*10*) are HIV-1 specific. We found that POLE3 and CAF1 are specific for retrotranscribed uHIV-1 DNA. Neither POLE3 KD nor CAF1 KD affected transfected HIV-1 molecular clone or reporter plasmid DNA under the control of either the HIV-1 or CMV promoter, while SMC5 KD and SLF2 KD enhanced this expression. Two findings suggest the specificity of POLE3 toward linear uHIV-1 DNA. First, uHIV-1 DNA repression by POLE3 was not affected by the absence of 2-LTR circles (Fig. 4, F to I). Second, POLE3 KD had no effect on luciferase activity driven by the LTR-luciferase reporter plasmid (Fig. 4, B to E). However, we cannot firmly exclude the possibility that POLE3 also regulates expression from circular vDNA. Identifying the determinants that can be located within the primary sequence or structures generated during the conversion of the positive (+) sense RNA genome into double-stranded DNA (gaps, nicks, and Flap) that govern this specificity is important.

After completion of reverse transcription, lvDNA can either undergo circularization to form 2-LTR and 1-LTR circles whose stability depends on the cell proliferation status (*29*, *30*) or serve as a template for viral integration into the host genome, an event required for vDNA maintenance and persistence (*1*, *2*). Selective targeting of uHIV-1 DNA species by specific host factors may differentially affect viral replication. uHIV-1 DNA circles may express early viral genes such as Tat to ensure rapid and robust transcription immediately after integration (*1*, *31*, *32*). In support of this hypothesis, HIV-1 evolved through VPR to overcome the transcriptional silencing imposed by SMC5/SMC6/SLF2 (*9*). In contrast, transcription from the linear form, which serves as a template for the viral integration into the host genome required for vDNA maintenance and persistence, may be detrimental to HIV-1 replication by reducing the efficiency of vDNA integration into the host genome due to its loading with the transcriptional machinery. Thus, by repressing transcription from linear uHIV-1 DNA, POLE3 would favor its integration into the host genome. In addition, POLE3 may interfere with the conversion of linear uHIV-1 DNA into circular forms to ensure its integration into the host genome. Increased abundances of total HIV-1 DNA and 2-LTR circles were observed upon POLE3 KD in cells infected with the IN mutant virus (Fig. 2, N and O) but not in those infected with the integration-competent virus (Fig. 5, B and C). Unfortunately, we were unable to assess the impact of POLE3 KD on the stability of linear uHIV-1 DNA due to the lack of a sensitive and quantitative method. However, we found that HIV-1 replication kinetics were impaired upon loss of POLE3. This finding suggests that transcriptional silencing imposed by POLE3 is beneficial for viral replication and may explain why HIV-1 did not evolve countermeasures to overcome the repressive activity of POLE3.

How does POLE3 facilitate HIV-1 replication? We foresee two possibilities that are not mutually exclusive. First, by increasing the efficiency of HIV-1 DNA integration, and second, by playing a role in preventing the sensing of uHIV-1 DNA, POLE3 helps to prevent the establishment of an antiviral state mediated by type I IFN. Thus, POLE3 joins the arsenal of viral and host proteins used by HIV-1 to escape the optimal innate immune response. However, inhibition of POLE3 alone is unlikely to lead to optimal induction of type I IFN as achieved by viral core disruption, which exposes several pathogen-associated molecular patterns to establish an efficient antiviral state (*26*, *27*). Further experiments are needed to determine the contributions of both functions of POLE3 to viral replication.

Understanding the establishment of chromatin on newly synthesized retroviral DNA and identifying the host factors involved in its transcriptional silencing are important not only for obtaining a better understanding retrovirus biology but also for establishing retroviruses as a unique and reliable model for studying the establishment of chromatin and its dynamics (*33*–*36*).

## MATERIALS AND METHODS

### Cells

SupT1 cells were grown in RPMI 1640 medium supplemented with 10% heat-inactivated fetal bovine serum (FBS), penicillin (100 U/ml), and streptomycin (100 μg/ml). 293T, HeLa, HeLa-P4, and TZM-bl cells were grown in Dulbecco’s modified Eagle’s medium (DMEM) supplemented with 10% FBS, 1 mM sodium pyruvate, and antibiotics. RPE1-hTERT Flag-Cas9 *TP53^−/−^* cells (WT and POLE3 KO) were provided by D. Durocher (*18*). RPE1 cells were grown in DMEM supplemented with 10% FBS, 1 mM UltraGlutamine, 1× nonessential amino acids, antibiotics, and blasticidin (2 μg/ml). Nalm-6 (*Lig4* +/+ or −/−) cells were provided by O. Delelis (*23*). These cells were cultured in RPMI 1640 medium supplemented with 10% FBS and antibiotics. HeLa-pl376 cells are HeLa cells harboring integrated HIV-1 expressing the luciferase reporter gene and lacking the *env* and *Tat* genes. All cells were cultured at 37°C in 5% CO_2_.

## Primary CD4^+^ T cell isolation, culture, and activation

Human primary CD4^+^ T cells were purified as described previously (*3*). Briefly, primary CD4^+^ T cells were isolated from non–HIV-infected individuals with RosetteSep Human CD4 T Cell Enrichment Cocktail (STEMCELL Tech) and purified by the Ficoll gradient method (Eurobio). CD4^+^ T cells were stimulated with PHA (0.5 μg/ml; Sigma-Aldrich) and IL-2 (50 U/ml; Roche) in RPMI 1640 medium supplemented with 10% heat-inactivated FBS, 1× MEM Non-Essential Amino Acids Solution, 2 mM UltraGlutamine, 1 mM sodium pyruvate, penicillin (100 U/ml), and streptomycin (100 mg/ml). Two days later, the culture medium was replaced with medium containing IL-2 (20 U/ml; Roche).

### Generation of POLE3 or POLE4 KD or KO SupT1, HeLa, and HeLa-P4 cells

POLE3- and POLE4-depleted cells were generated using the Alt-R CRISPR-Cas9 System [Integrated DNA Technologies (IDT)]. The CRISPR RNAs (crRNAs) targeting the *POLE3* gene (GATCCTGGTGATCACGGCAT, Hs.Cas9.POLE3.1.AB, IDT) and the *POLE4* gene (AGATCCCGACGTGACGCTAG, HS.Cas9.POLE4.1. AA, IDT), as well as the nontargeting (NT) crRNA (CRISPR Negative Control crRNA, IDT), were annealed with ATTO-550–labeled universal tracrRNAs (Alt-R CRISPR-Cas9 tracrRNA ATTO 550, IDT) and subsequently incubated with recombinant Cas9 (Alt-R S.p. HiFi Cas9 Nuclease V3, IDT) to form RNP complexes. For depletion in SupT1 cells, RNP complexes were electroporated together with carrier DNA (Alt-R Cas9 Electroporation Enhancer, IDT) using an Amaxa 4D-Nucleofector X Unit (Amaxa SF Cell Line 4D-Nucleofector X Kit S, Lonza). The electroporation setting was SF Solution CA-137 Pulse. For depletion in HeLa and HeLa-P4 cells, RNP complexes were reverse-transfected into HeLa cells using RNAiMAX transfection reagent (Thermo Fisher Scientific). Twenty-four (SupT1) or 48 (HeLa and HeLA-P4) hours later, ATTO550-positive cells were sorted by a cell sorter (BD FACSMelody Cell Sorter, BD Biosciences) and cultured in the appropriate medium. To obtain a single KO cell clone, the sorted cells were diluted and spread in 96-well plates. The expression levels of POLE3 and POLE4 in the KO cells were measured by Western blotting, and the sequences in the clones were determined after PCR amplification from genomic DNA using POLE3- and POLE4-specific primers (PolE3-forward-cr: TGGCGAGAAGCAGAGAAATGG; PolE3-reverse-cr: CAGACTCCCTCTTGTTTGTAAGGC; PolE4-forward-cr: AGAGGAGGAGGGACCTG; PolE4-reverse-cr: GGGCAGAAAGGGAGAAGAAA; PolE4 KO-1 forward: CAGATTTCGCTGCCTCCTTG; PolE4 KO-1 reverse: CAGAGAAGAACGTGGCAATAG) and subcloning using the Zero Blunt TOPO PCR Cloning Kit for Subcloning (Thermo Fisher Scientific) (table S6).

### Overexpression of POLE3 in HeLa and HeLa-P4 cells

POLE3 KO HeLa cells with exogenous expression of the POLE3 WT, POLE3 F44D, or POLE3 ∆C protein and POLE3 KO HeLa-P4 cells with exogenous expression of the POLE3 WT protein were constructed using previously described MMLV-based retroviral constructs (*37*, *38*) containing a bicistronic transcriptional unit allowing the expression of a selection marker [puromycin resistance gene (pOZ-puro)]. Cells expressing the different constructs were selected in medium supplemented with 1 μg/ml (HeLa cells) or 7.5 μg/ml (HeLa-P4) puromycin for 2 days.

### Plasmids

The pHIV-Luc IN^wt^, pHIV-Luc IN^D116A^, pHIV-Luc Tat^−^ (Env^−^, full-length HIV-1 vector expressing firefly luciferase via the nef gene), and ps/DNA5/FRT/LTR HIV-1–luciferase (pLTR-Luc) plasmids were provided by S. Emiliani. pNL4-3 and pNL4.3Luc.R-.E- were obtained from the NIH AIDS Reagent Program. pNL4.3.Luc.R-.E-.IN^D64A^ was provided by S. Goff. pNL4.3.Luc.R+.E-.IN^D64A^ (VPR^+^) was constructed from the pNL4.3.Luc.R-. E- IN^D64A^ plasmid by site-directed mutagenesis. Briefly, an AgeI-SalI fragment encompassing the IN and VPR genes was amplified using the primers AAGAACCGGTACATGGAGTGTATTATGACC (forward) and CTATGTCGACACCCAATTCTGAAATGGATAAACAG (reverse) and inserted into the vector provided in the Zero Blunt TOPO PCR Cloning Kit for subcloning (Thermo Fisher Scientific). The VPR gene was restored by site-directed mutagenesis using the QuikChange Lightning Site-Directed Mutagenesis Kit (Agilent) with the following primers: GGACACTAGAGCTTTTAGAGGAACTTAAGAGTGAAGCTGTTAG (forward) and CTAACAGCTTCACTCTTAAGTTCCTCTAAAAGCTCTAGTGTCC (reverse). After sequencing, the mutated CPR^+^ fragment was subcloned back into the pNL4.3.Luc. E- IN^D64A^ pCMVdeltaR8.2 packaging plasmid obtained from D. Trono (Addgene plasmid 12263). The pMD2-G VSV-G envelope plasmid was previously described (*39*). pCMV-intron, pCMV-intron IN^D184A^, and pCNA-luc were provided by S. Goff (*28*). The plasmids encoding POLE3, POLE3 F44D, and POLE3 ∆C were provided by S. J. Boulton and R. Bellelli (*7*). The plasmid encoding HIV Flag-Tat was previously described (*40*). The pCMV-Luc plasmid was previously described (*41*).

### Viral production

Viral stocks were produced in 293T cells (2 × 10^6^ cells/10-cm plate) using the phosphate calcium transfection method. HIV replication–defective viral particles were generated by cotransfection of 8 μg of the pHIV-Luc IN^wt^ or IN^D116A^ plasmid and 2 μg of pMDG2. HIV replication–competent viral particles were generated by transfection of 8 μg of pNL4.3. VSV-G pseudotyped NL4.3 was generated by cotransfection of 8 μg of pNL4.3 and 2 μg of PMD2-G. MLV replication–defective viral particles were generated by cotransfection of 3 μg of pCMV-intron (MLV gag-pol) or pCMV-intron IN^D184A^ (MLV gag-Pol with IN mutation) along with 6 μg of pCNA-luc (MLV vector expressing firefly luciferase) and 2 μg of pMDG2. Sixteen hours later, the medium was replaced with DMEM supplemented with 1% FBS. After 48 hours of transfection, the supernatants were harvested, filtered (0.45 μm), and concentrated by ultracentrifugation through a 20% sucrose cushion. The produced replication-competent NL4.3 viral particles were not concentrated, and VSV-G pseudotyped NL4.3 viral supernatants were concentrated with a Lenti-X concentrator (Takara Bio). Viruses were treated with DNase (100 U/ml; Turbo DNase, Thermo Fisher Scientific) at 37°C for 1 hour. HIV production was quantified by measurement of the p24 antigen concentration by enzyme-linked immunosorbent assay (ELISA) (ZeptoMetrix), and the MOI was determined in SupT1 and HeLa cells.

### PICh

The PICh experimental procedure was adapted from Déjardin *et al.* (*6*, *42*). A total of 4.5 × 10^9^ SupT1 cells were infected with the HIV-Luc virus at an MOI of 2 for 9 hours. A total of 4.5 × 10^9^ noninfected SupT1 cells were used as the control. Cells were subjected to crosslinking with 3.6% formaldehyde for 45 min at room temperature and were then washed four times in PBS by centrifugation at 3200*g* for 10 min at 4°C. Cell pellets were frozen in liquid nitrogen and stored at −80°C or used immediately for PICh. Cells were equilibrated in sucrose buffer [10 mM Hepes NaOH (pH 7.9), 300 mM sucrose, 1% Triton X-100, 2 mM MgOAc, and 100 mM NaCl] and dounced 20 times with a tight pestle. Cells were then washed in PBS and resuspended in PBS-0.5% Triton X-100 supplemented with RNase A (5 ml of PBS-0.5% Triton X-100 + 15 U RNase/ml of solubilized pellet) and incubated for 2 hours at room temperature and overnight at 4°C on a rotating wheel. The pellets were then washed in PBS and aliquoted as 1.5 ml of solubilized pellet per 15-ml tube. The pellets were then resuspended in MNase digestion buffer [20 mM Hepes-NaOH (pH 7.9), 5 mM MgCl_2_, 5 mM CaCl_2_, and 70 mM KCl in PBS-0.5% Triton X-100]. The pellets were preheated at 37°C, and MNase (500 U/1.5 ml solubilized pellet) was added for a 30 min incubation with shaking at 500 rpm. MNase digestion was stopped by adding EGTA to a final concentration of 5 mM. The pellets were then washed once with PBS + 5 mM EGTA and three times with PBS and were then equilibrated in LB4 buffer [50 mM tris-HCl (pH 8), 200 mM NaCl, 20 mM EDTA, and 1% SDS] and sonicated (Misonix S-400 sonicator) using the following parameters: power setting 7 (36 to 45 watts), 15-s constant pulse, 45-s pause, for a total processing time of 1 min and 30 s. The samples were then incubated at 58°C for 5 min with shaking at 500 rpm and centrifuged at 16,000*g* for 10 min at room temperature to remove all debris. The chromatin was then ready to use for PICh. The chromatin was first precleared with Ultralink Streptavidin Agarose beads preequilibrated with LB4 buffer overnight at 4°C on a rotating wheel. The precleared chromatin was recovered by passage through Sephacryl S-400-HR resin. Sixty milligrams of chromatin was used for hybridization with probes targeting the first 1.5 kb of the HIV genome (LTR-gag). These probes were designed as follows: desthiobiotin/spacer18/spacer18/2′-fluoro-RNA/DNA sequence (synthesized by the Keck Foundation, Yale University). Fifteen microliters of a 100 μM stock mixture was added per 10 mg of chromatin (table S1). Hybridization was conducted as follows: 25°C for 3 min, 82°C for 5 min, 37°C for 1 hour, 60°C for 3 min, 37°C for 30 min, 60°C for 3 min, and 37°C for 30 min, holding at 25°C. The hybridized chromatin was then centrifuged at 16,000*g* for 15 min, and the supernatant was diluted with one volume of Milli-Q water and added to MyONE C1 magnetic beads (900 μl of beads per 15 μl of probe) preequilibrated in LB3JD buffer [10 mM Hepes-NaOH (pH 7.9), 100 mM NaCl, 2 mM EDTA (pH 8), 1 mM EGTA (pH 8), 0.2% SDS, and 0.1% Sarkosyl] for 1 hour and 30 min30 at room temperature on a rotating wheel. The beads were washed five times with 10 ml of LB3JD per 900 μl of beads, transferred to a 5-ml tube, and washed one more time with 5 ml of LB3JD. The beads were then washed once with 5 ml of low-salt LB3JD buffer [10 mM Hepes-NaOH (pH 7.9), 30 mM NaCl, 2 mM EDTA (pH 8), 1 mM EGTA (pH 8), 0.2% SDS, and 0.1% Sarkosyl] for 5 min at 42°C with shaking at 1000 rpm. Last, the beads were resuspended in elution buffer (12.5 mM d-biotin in LB3JD buffer) and incubated with shaking at 37°C for 30 min and at 65°C for 10 min. For protein analysis, the input and eluate were precipitated using trichloroacetic acid (17% final concentration) for 50 min on ice, and the precipitated proteins were washed two times in ice-cold acetone. The input and eluate fractions for PICh were resuspended in 400 and 80 μl, respectively, of crosslinking reversal solution [250 mM tris-HCl (pH 8.8), 2% SDS, and 1.43 M 2-mercaptoethanol] and incubated for 25 min at 99°C. Protein samples were stored at −80°C. For protein analysis, proteins were separated using 4 to 12% bis-tris acrylamide precast gels (Invitrogen), and the gels were subjected to silver staining (Silver Quest Kit, Invitrogen) or colloidal blue staining according to the manufacturer’s instructions or subjected to Western blot analysis using an anti-IN antibody. Colloidal blue-stained gels were used for mass spectrometry analysis (Taplin Mass Spectrometry Facility). For DNA analysis, the input (0.04%), FT (0.02%) and eluate (0.1%) were incubated at 65°C overnight (1100 rpm) in crosslinking reversal solution [250 mM tris-HCl (pH 8), 150 mM NaCl, and 0.5% SDS]. RNase A (0.2 mg/ml final concentration) was added for a 1-hour incubation at 37°C (1100 rpm), and proteinase K (0.2 mg/ml final concentration) was then added for a 2-hour incubation at 65°C (1100 rpm). After phenol-chloroform extraction and ethanol precipitation, the DNA pellet was resuspended in water, and qPCR was performed using β-globin, α-satellite, and Nuc0 and Nuc1 primers. Standard curves generated using the input DNA fraction were used to quantify β-globin and α-satellite regions in the eluates. A standard curve generated using the HIV-Luc molecular clone was used to quantify HIV-1 DNA using the Nuc0 and Nuc1 primers. Values are expressed relative to the input fraction (table S8).

### Plasmid pull-down assay

The plasmid pull-down assay was modified from Ide and Dejardin (*43*). A total of 1.5 μg of the pLTR-luc plasmid was linearized by KpnI-PmeI digestion. The linearized plasmid was divided into two aliquots and resuspended in LB3JD buffer containing two different sets of probes (0.1 μM final concentration). One set was composed of probes targeting the LTR region (SH1-SH15), and the other set was composed of probes targeting the Gag region (SH16-SH30). Hybridization was conducted as follows: incubation at 93°C for 2 min, decreasing to 37°C with a ramp of 1°C/min, 37°C for 30 min. Then, 75 μl of MyONE C1 magnetic beads preequilibrated in LB3JD buffer was added to the DNA, and the mixture was incubated for 1 hour and 30 min at room temperature on a rotating wheel. The beads were then immobilized on a magnetic stand, and the supernatant was collected as the FT. The beads were washed five times with LB3JD buffer, resuspended in elution buffer (12.5 mM d-biotin in LB3JD buffer) and incubated for 15 min at 65°C with shaking at 500 rpm. One-tenth volumes of the input, FT, and eluate fractions were analyzed by agarose gel electrophoresis, and the gels were then stained with ethidium bromide. Band intensities were assessed using Image Lab software (Bio-Rad). Values are presented relative to the input in three independent experiments.

### siRNA screening

siRNA screens were performed in 96-well plates using custom siRNA libraries based on the candidates identified by the mass spectrometry in the eluates used for the PICh experiment on the uvDNA. siRNA libraries were purchased from Horizon Discovery (Dharmacon). For the screen referenced in Fig. 1D, the siRNA library was composed of 455 siRNAs organized as pools of 4 siRNAs. Negative control siRNAs (NT, GAPDH, and luciferase GL3) were added (table S7) in random wells on each plate. HeLa cells (10,000 cells per well) were reverse-transfected with 40 nM siRNA (final concentration) using Lipofectamine RNAiMAX reagent (Thermo Fisher Scientific) and infected 48 hours later with the VSV-G pseudotyped HIV-Luc IN^D116A^ virus at an MOI of 0.2 for 48 hours. For the screen referenced in Fig. 1F, the siRNA library for the 24 factors was composed of four individual siRNAs (table S8). The screen was performed as previously described with a final siRNA concentration of 25 nM. Luciferase activity was measured using BrightGlo luciferase assay reagent (Promega), and luminescence was measured with a TriStar LB 941 multimode plate reader (Berthold Technologies). The impact of the siRNAs on cell viability was assessed 48 hours posttransfection using CellTiter-Glo (Promega). siRNAs inducing a decrease in cell viability of 25% or more were removed from subsequent analyses. Each siRNA screen was performed in triplicate in two independent experiments. Values are presented as the mean luciferase activity relative to that in control siNT-transfected cells.

## Luciferase assay

HeLa cells were reverse-transfected with 25 nM siRNA (final concentration). Forty-eight hours later, the cells were infected with VSV-G pseudotyped HIV-Luc IN^D116A^ at an MOI of 0.5. After 3 hours, the cell culture medium was replaced. Cells were lysed in passive lysis buffer (Promega) at 48 hpi. Insoluble proteins were pelleted by centrifugation at 16,300*g*, and the supernatant was collected and analyzed with a luciferase assay system (Promega). Luminescence was measured with a TriStar LB 941 multimode plate reader (Berthold Technologies) and normalized to the protein concentration as measured with a Pierce BCA Protein Assay Kit (Thermo Fisher Scientific).

RPE1-hTERT Flag-Cas9 TP53^−/−^ WT and POLE3 KO cells, HeLa POLE3 KO and POLE4 KO cells, and HeLa POLE3-reconstituted cells were infected with VSV-G pseudotyped HIV-Luc IN^D116A^ at an MOI of 0.5. After 3 hours, the cell culture medium was replaced. Cells were lysed in passive lysis buffer at 48 hpi. Luminescence was measured as described above.

For experiments using Nalm-6 (*Lig4* +/+ or −/−) cells, cells were electroporated with 3.3 μM NT or POLE3 siRNA using an Amaxa 4D-Nucleofector X Unit (SF Cell Line 4D-Nucleofector X Kit, Lonza). The electroporation setting was CV-104. Two days later, the cells were infected with VSV-G pseudotyped HIV-Luc IN^D116A^ at an MOI of 0.05. After 3 hours, the cell culture medium was replaced. Cells were lysed in passive lysis buffer at 48 hpi. Luminescence was measured as described above.

For experiments using primary CD4^+^ T cells, CD4^+^ T cells were activated by PHA–IL-2 for 2 days and were then cultured in medium supplemented with IL-2 for 1 day. Activated CD4^+^ T cells were then electroporated with 5 μM NT or POLE3 siRNA using an Amaxa 4D-Nucleofector X Unit (P3 Primary Cell 4D-Nucleofector X Kit, Lonza). The electroporation setting was Stimulated T cells (P3 solution, EO115 pulse). Two days later, the cells were infected with VSV-G pseudotyped HIV-Luc IN^D116A^ at an MOI of 0.5. After 3 hours, the cell culture medium was replaced. Cells were lysed in passive lysis buffer at 48 hpi. Luminescence was measured as described above.

### Quantification of vDNA

Cells were transfected with siRNAs and infected as described above. Cells were collected at 9 or 48 hpi, and DNA was extracted using the DNeasy Blood and Tissue Kit (QIAGEN). For kinetic analysis, SupT1 cells were infected with VSV-G pseudotyped HIV-Luc IN^wt^ at an MOI of 0.8 as an infectious pool. Three hours later, the cells were washed three times in PBS and aliquoted into six wells. Cells were collected at 3, 6, 9, 12, 24, and 48 hpi, and DNA was extracted using the DNeasy Blood and Tissue Kit (QIAGEN). Total HIV DNA and 2-LTR circles were quantified by qPCR. Integrated provirus was quantified by Alu PCR followed by qPCR (*3*, *44*). For experiments in HeLa P4 cells, integrated provirus was quantified as described previously (*45*), and the second round of qPCR was performed using primers for DHS. The vDNA abundance was normalized to the β-globin abundance. The primers used for vDNA quantification are listed in table S9.

### Quantification of IFNβ and IFN-related gene expression

Primary CD4^+^ T cells isolated from healthy donors were activated with PHA-Il2 and transfected with siNT or POLE3 siRNA. Forty-eight hours later, the cells were infected with VSV-G pseudotyped HIV-Luc IN^wt^ or IN^D116A^ for 24 hours. Cells were harvested, and RNA was extracted using an RNeasy Plus Mini Kit (QIAGEN) according to the manufacturer’s instructions. cDNA synthesis was performed using SuperScript IV enzyme (Thermo Fisher Scientific). IFNβ, ISG15, IFIT1, IFIT2, and GAPDH mRNA expression was quantified by qPCR using specific primers (table S9).

### ChIP assays

ChIP assays for histones and RNAPII were performed as described previously (*3*). Briefly, HeLa cells were reverse-transfected with 25 nM siRNA (final concentration). Forty-eight hours later, the cells were infected with VSV-G pseudotyped HIV-Luc IN^D116A^ at an MOI of 1. After 3 hours, the cell culture medium was replaced. For experiments using primary CD4^+^ T cells, activated CD4^+^ T cells were electroporated as described above and infected with VSV-G pseudotyped HIV-Luc IN^D116A^ at an MOI of 0.5. After 3 hours, the cell culture medium was replaced. Cells were collected at 48 hpi and fixed with 1% formaldehyde for 10 min. ChIP assays were performed using the SimpleChIP Plus Enzymatic Chromatin IP Kit (Cell Signaling Technology) according to the protocol provided by the manufacturer. Chromatin treated with 10 to 15 μg of MNase was reacted with 4 μg of anti-H3, anti-H3Ac, anti-RNAPII, and anti-H1.2 antibodies (table S10). For the POLE4 ChIP assay, POLE4 KO HeLa cells were infected with VSV-G pseudotyped HIV-Luc IN^D116A^ at an MOI of 1. After 3 hours, the cell culture medium was replaced. Cells were collected at 48 hpi and fixed with ChIP Cross-link Gold (Diagenode) for 40 min in the presence of 1 mM MgCl_2_ before fixation with 1% formaldehyde for 15 min. The fixation reaction was quenched with glycine, and the cells were washed with PBS. After washing, the cells were treated with lysis buffer 1 [50 mM Hepes-KOH (pH 7.5), 140 mM NaCl, 1 mM EDTA, 10% glycerol, 0.5% NP-40, 0.25% Triton X-100, 1 mM phenylmethylsulfonyl fluoride (PMSF), and protease inhibitor cocktail] for 10 min on ice. After centrifugation, the nuclear pellets were washed with lysis buffer 2 [10 mM tris-HCl (pH 8.0), 200 mM NaCl, and 1 mM EDTA]. After centrifugation, nuclei were resuspended in shearing buffer D3 [10 mM tris-HCl (pH 7.4), 1 mM EDTA, 0.1% SDS, 1 mM PMSF, and protease inhibitor cocktail] and incubated for 15 min on ice. Chromatin was fragmented by sonication with a Covaris S220 focused ultrasonicator and was then diluted with the same volume of dilution buffer [10 mM tris-HCl (pH 7.4), 300 mM NaCl, 1 mM EDTA, 2% Triton X-100, 1 mM PMSF, and protease inhibitor cocktail]. Immunoprecipitation was performed using a ChIP-IT High Sensitivity Kit according to the protocol provided by the manufacturer (Active Motif). Thirty micrograms of sonicated chromatin was reacted with 4 μg of an anti-POLE4 antibody (table S10). qPCR was performed using specific primers (table S9).

### CUT&RUN assay

HeLa cells were reverse-transfected with 25 nM POLE3 siRNA (final concentration). Forty-eight hours later, the cells were infected with VSV-G pseudotyped HIV-Luc IN^D116A^ at an MOI of 1. After 3 hours, the cell culture medium was replaced. Cells were collected at 48 hpi. CUT&RUN was performed on 0.25 million permeabilized cells with 1 μg of an anti-POLE3 antibody using a CUT&RUN assay kit (Cell Signaling Technology) according to the protocol provided by the manufacturer. Input DNA was extracted from 0.25 million permeabilized cells using a Gentra Puregene kit (QIAGEN). qPCR was performed using specific primers (table S9).

## Nascent RNA IP

HeLa cells were transfected with siRNAs and infected with VSV-G pseudotyped HIV-Luc IN^D116A^ at an MOI of 0.5 as described above. After 3 hours, the cell culture medium was replaced. Forty-eight hours after infection, the cells were treated with 0.5 mM 5-ethynyl uridine (EU) for 30 min and washed twice with PBS. Total RNA was extracted from EU-treated cells with TRIzol reagent (Thermo Fisher Scientific), treated with DNase I (TURBO DNA-free Kit, Invitrogen), and subjected to nascent RNA capture using the Click-iT Nascent RNA Capture Kit (Thermo Fisher Scientific). RT-qPCR was then performed. Briefly, 1 μg of total RNA was biotinylated by a click reaction, and 0.5 μg of biotinylated RNA was bound to Dynabeads MyOne Streptavidin T1 magnetic beads. Bead-bound RNA was subjected to RT using SuperScript IV Reverse Transcriptase (Thermo Fisher Scientific) with Random Hexamer Primer (Thermo Fisher Scientific). qPCR was performed using primers specific for the luciferase gene in the HIV-Luc IN^D116A^ genome (Luc 85) and for Rhot2 in the human genome (table S9). Nascent viral RNA expression was normalized to Rhot2 expression.

## HIV-1 replication assay

For SupT1 cells, parental and POLE3 KD cells (10^6^) were infected with 1.2 μg (MOI #1) or 240 ng (MOI #2) of replication-competent HIV WT (NL4.3) virus. After 3 hours, the infected cells were washed twice with PBS and were then cultured in 1 ml of medium. Every 3 days, half of the culture supernatant was collected for p24 ELISA, and an equal amount of complete medium was added to the remaining half of the cell culture medium. For HeLa-P4 cells, control and POLE3 KO cells were seeded in a six-well plate (250,000 cells per well) and infected overnight with 14 (MOI #1) or 2.8 ng (MOI #2) NL4.3 viral P24. Cells were then washed to remove input virus. Every 3 days, half of the culture supernatant was transferred to a new six-well plate, and the other half was collected for p24 ELISA. Cells were trypsinized, and equal amounts of cells were added to the culture supernatant. HIV-1 spreading was quantified by measuring the p24 concentration in the culture medium using the HIV-1 p24 Antigen ELISA 2.0 Kit (ZeptoMetrix, 0801008).

### HIV-1 production and infectivity assays

For the HIV production assay, HeLa cells were infected with VSV-G pseudotyped HIV-Luc IN^wt^ for 24 hours and transfected with siRNAs. Two days later, viral production was assessed by measuring the P24 antigen concentration in the supernatant by ELISA (ZeptoMetrix), and a luciferase assay was performed as described above.

For the viral infectivity assay, HeLa siNT-transfected and POLE3 KO cells were infected with VSV-G pseudotyped NL4.3 (env^+^) at an MOI of 0.5. After 3 hours, the cell culture medium was replaced. Forty-eight hours after infection, supernatants were collected, and the p24 antigen concentration was measured by ELISA (released CA). The infectivity of the released viruses was determined by infection of TZM-bl reporter cells in 96-well plates. Twenty-four hpi, luciferase activity was measured using Bright Glo luciferase assay reagent (Promega). HIV infectivity was represented by the ratio of the luminescence intensity to the quantity of p24 CA measured in the supernatant.

## Supplementary Materials

### This PDF file includes:

Figs. S1 to S6 Legends for tables S1 to S10 References
