# Supplementary material for: POLE3 is a repressor of unintegrated HIV-1 DNA required for efficient virus integration and escape from innate immune sensing
Source: Sci Adv. 2023 Nov 3;9(44):eadh3642. doi: 10.1126/sciadv.adh3642 (PMC10624344; doi:10.1126/sciadv.adh3642)
Supplement: Supplementary file 1 — Figs. S1 to S6 Legends for tables S1 to S10 References [file sciadv.adh3642_sm.pdf]

Science Advances
AAAS

Supplementary Materials for

POLE3 is a repressor of unintegrated HIV-1 DNA required for efficient virus integration and escape from innate immune sensing

Suzie Thenin-Houssier *et al.*

Corresponding author: Monsef Benkirane, monsef.benkirane@igh.cnrs.fr

Sci. Adv. **9**, eadh3642 (2023)
DOI: 10.1126/sciadv.adh3642

The PDF file includes:

Figs. S1 to S6
Legends for tables S1 to S10
References

Other Supplementary Material for this manuscript includes the following:

Tables S1 to S10

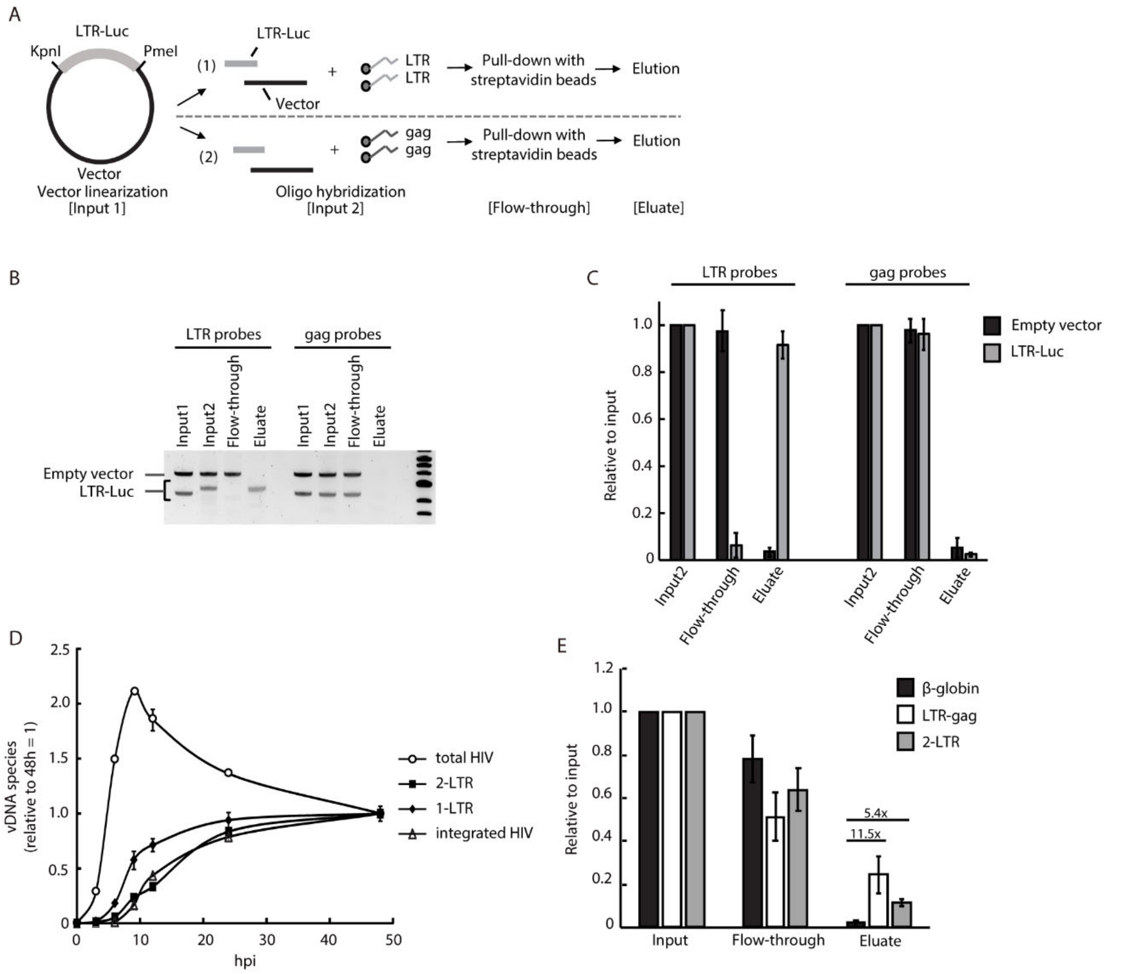

Fig S1. Optimization of the PICh method for the isolation of uHIV-1 DNA in vitro and in infected cells.

(A) Outline of the procedure for the plasmid pull-down assay using the LTR-luc plasmid. Linearized plasmid was mixed with PICh probes specific for the LTR (1) or Gag (2) linked to a spacer and desthiobiotin. After partial denaturation/hybridization cycles, hybrids were captured using streptavidin beads, washed extensively and eluted with biotin.

(B) Each fraction (input, FT, and eluate) was analyzed by agarose gel electrophoresis and EtBr staining.

(C) Quantification of target (LTR-luc) capture, presented as percent DNA relative to input 2.

(D) SupT1 cells were infected with VSV-G pseudotyped HIV-Luc at an MOI of 0.8. Cells were harvested at 3, 6, 9, 12, 24 and 48 hpi. Total HIV DNA, 2-LTR and 1-LTR circles, and integrated DNA were analyzed by qPCR. The data are presented as quantities relative to the corresponding values at 48 hpi. The mean values ± SDs of three independent experiments are plotted.

(E) Capture of genomic DNA from SupT1 cells infected with HIV-Luc for 9 hr. Genomic DNA was hybridized to probes designed for PICh, captured on streptavidin beads and eluted with biotin. qPCR analysis of the input, FT and eluate fractions using the indicated primers. The results are

presented as quantities relative to input. Fold enrichment of vDNA over β-globin is shown. The mean values ± SDs of four independent experiments are plotted.

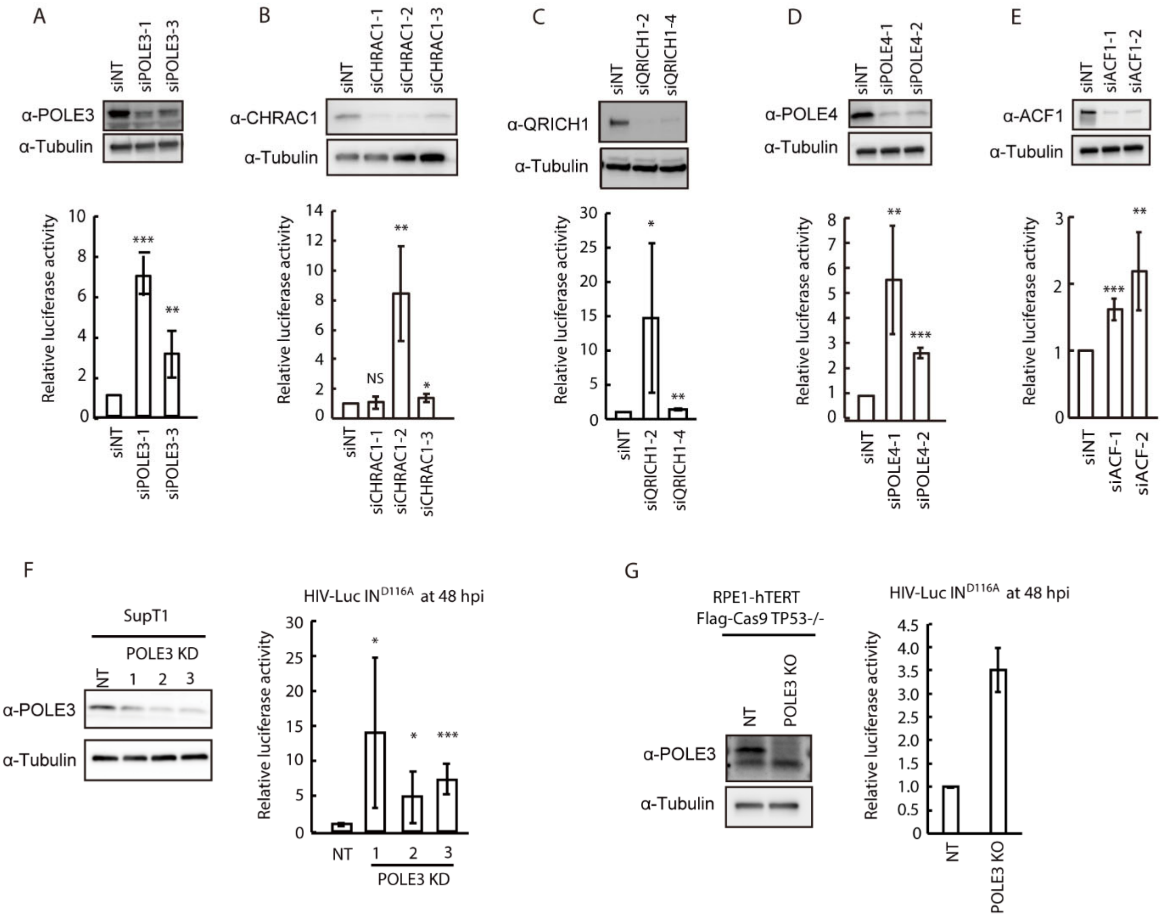

Fig S2. CHRAC1 and QRICH1 are not involved in uHIV-1 DNA silencing.

(A-E) Expression of POLE3 (A), CHRAC1 (B), QRICH (C), POLE4 (D) and ACF1 (E) proteins in cells treated with the indicated siRNAs was analyzed by immunoblotting using the indicated antibodies. Luciferase assays in KD cells were performed at 48 hpi. The results are presented as luciferase activity relative to that in NT siRNA-transfected cells, and the mean ± SD values of 2 independent experiments with triplicate samples are plotted.

(F) Impact of POLE3 depletion in SupT1 cells on uHIV-1 DNA silencing. POLE3 protein expression in POLE3 KO cells was analyzed by immunoblotting with the indicated antibodies. Luciferase assays in POLE3 KO cells were performed at 48 hpi. The results are presented as luciferase activity relative to that in NT sgRNA cells, and the mean ± SD values of two independent experiments with triplicate samples are plotted.

(G) Effect of POLE3 depletion on uHIV-1 DNA silencing in RPE-1-hTERT Flag-Cas9 TP53-/- cells. POLE3 expression in whole-cell extracts on the day of infection was analyzed by immunoblotting using an anti-POLE3 antibody. Luciferase assays were performed at 48 hpi. The results are presented as luciferase activity relative to that in nontargeting (NT) sgRNA cells, and the mean ± SD values of three independent experiments with triplicate samples are plotted.

* $P<0.05$, ** $P<0.01$, *** $P<0.001$; independent Student's t test.

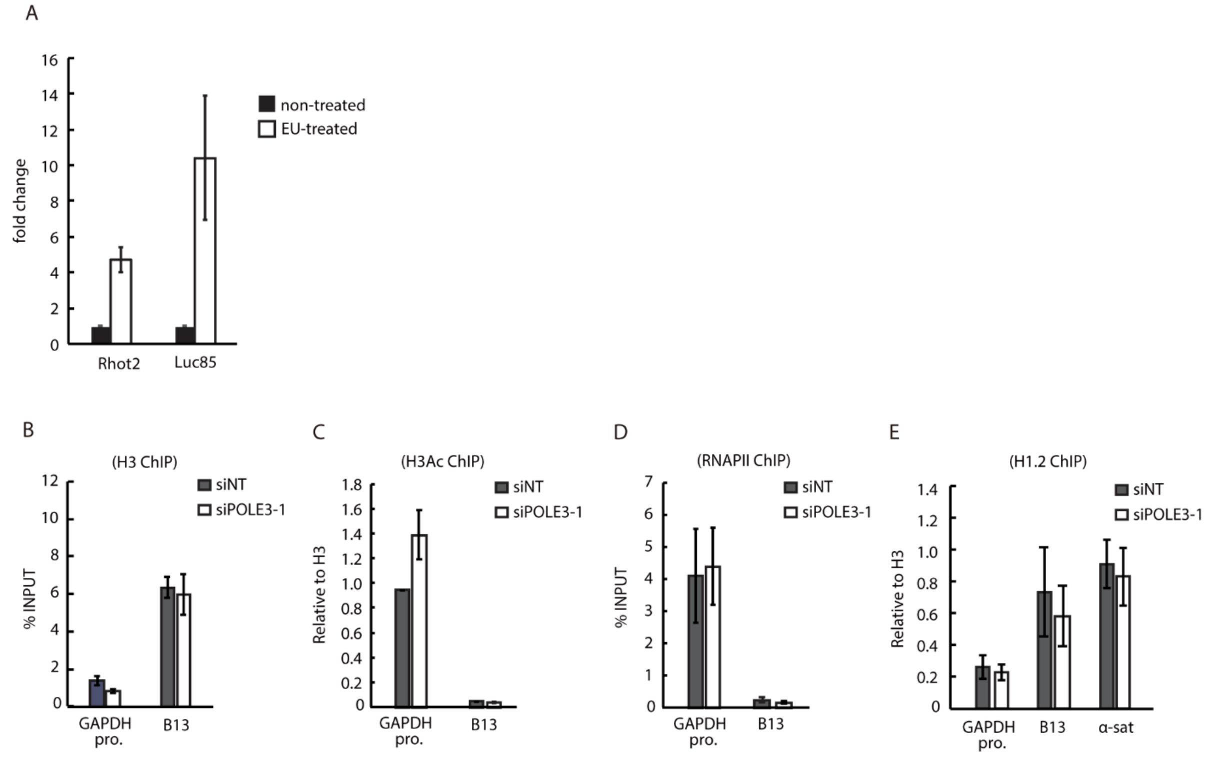

Fig S3. Nascent HIV RNA IP and ChIP qPCR assays in human genomic regions.
(A) Enrichment of EU-labeled RNAs synthesized in HeLa cells infected with VSV-G pseudotyped HIV-Luc IN^{D116A} at 48 hpi. RNA was extracted from HeLa cells treated with or without EU. Labeled RNAs were biotinylated and purified with streptavidin-conjugated beads. Enrichment of labeled RNAs is presented as the fold change with respect to unlabeled conditions. The mean ± SD values of 2 independent experiments with triplicate samples are plotted.
(B-E) ChIP assay using NT (gray) and POLE3 (white) KD HeLa cells infected with VSV-G pseudotyped HIV-Luc IN^{D116A}. ChIP assays were performed at 48 hpi using anti-H3 (B), anti-H3Ac (C), anti-RNAPII (D), and anti-H1.2 (E) antibodies; corresponding to Fig. 2F-I. qPCR analysis was performed using specific primers for the GAPDH promoter, B13, and α-satellite. The mean ± SD values of at least 3 independent experiments are plotted.

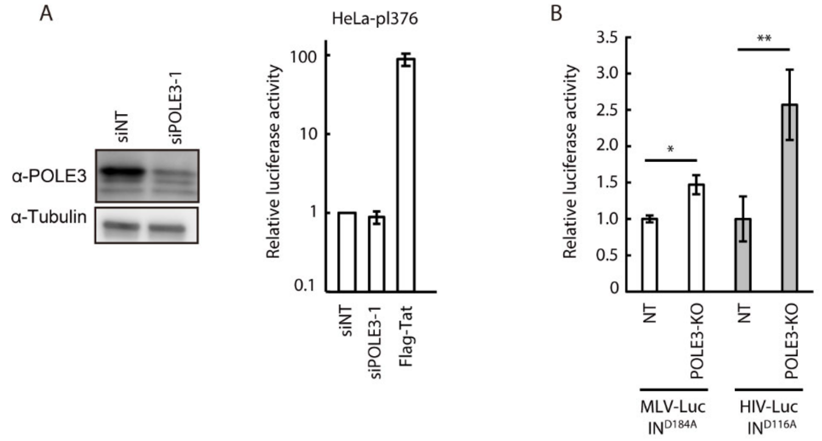

Fig S4. POLE3 has no impact on integrated HIV DNA and a marginal effect on uMLV DNA.

(A) Effect of POLE3 KD on integrated HIV DNA. POLE3 expression in whole-cell extracts was analyzed by immunoblotting using an anti-POLE3 antibody. Luciferase assays were performed using HeLa-pl376 cells (cells containing integrated HIV expressing luciferase and lacking the *env* and *tat* genes) treated with NT or POLE3 siRNA. As a positive control, cells were transfected with a plasmid encoding Flag-Tat. The results are presented as luciferase activity relative to that in NT siRNA-transfected cells, and the mean ± SD values of 2 independent experiments with triplicate samples are plotted.

(B) Effect of POLE3 KD on uMLV DNA. Luciferase assay in POLE3 KO HeLa-P4 cells infected with VSV-G pseudotyped MLV-Luc IN^{D184A} or HIV-Luc IN^{D116A} at 48 hpi. The results are presented as luciferase activity relative to that in NT sgRNA cells, and the mean ± SD values of three representative experiments in triplicate.

* P<0.05, ** P<0.01; independent Student's t test.

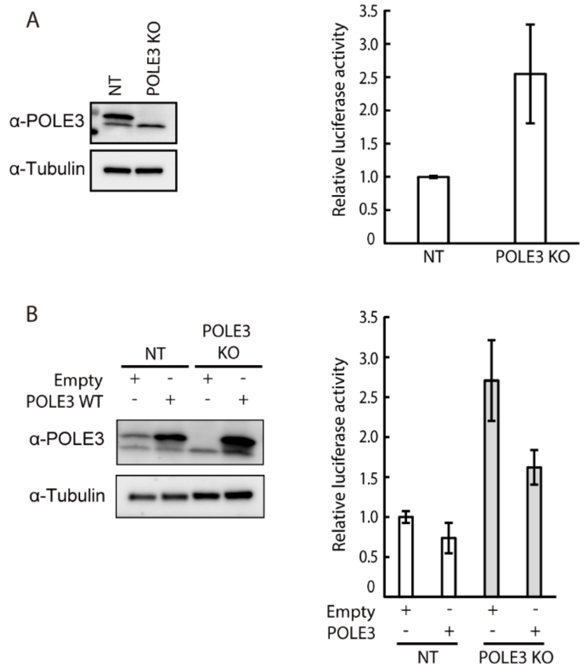

Fig S5. POLE3 depletion in HeLa-P4 cells.

(A) Impact of POLE3 KO in HeLa-P4 cells on uHIV-1 DNA silencing. POLE3 protein expression in POLE3 KO HeLa-P4 cells was analyzed by immunoblotting with the indicated antibodies. Luciferase assays were performed at 48 hpi. The results are presented as luciferase activity relative to that in NT sgRNA cells, and the mean ± SD values of at least 3 independent experiments with triplicate samples are plotted.

(B) Exogenous POLE3 expression in POLE3 KO cells silences uHIV-1 DNA transcription. POLE3 KO HeLa-P4 cells with stable exogenous expression of the POLE3 WT were infected with VSVG pseudotyped HIV-Luc IN^{D116A}. Whole-cell extracts were analyzed by immunoblotting with the indicated antibodies. Luciferase activity was measured at 48 hpi. The results are presented as luciferase activity relative to that in NT sgRNA cells in 3 independent experiments performed with triplicate samples.

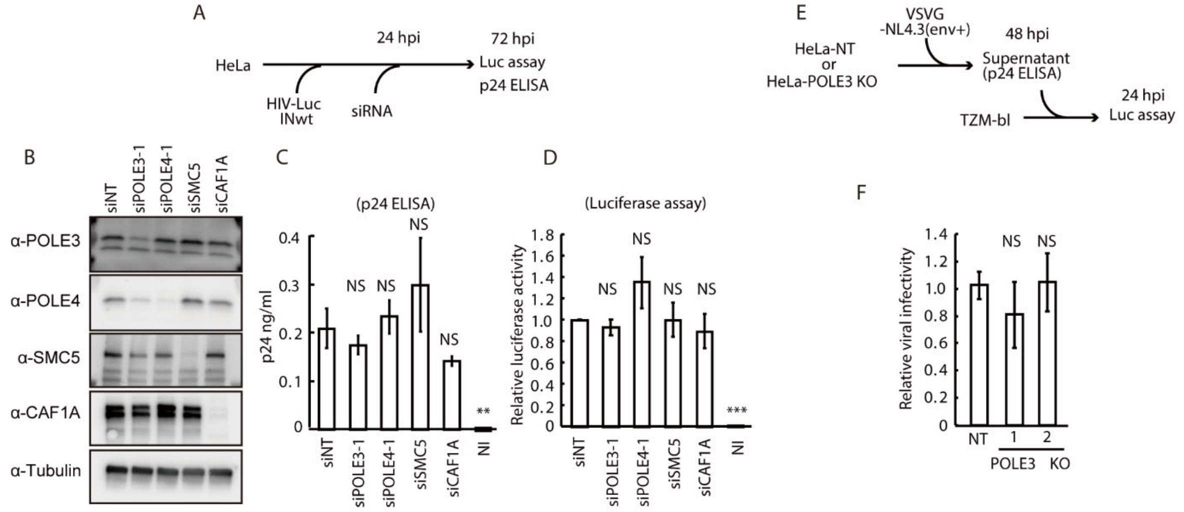

Fig S6. POLE3 depletion does not affect the late step of viral production.

(A-D) Effects of POLE3, POLE4, SMC5, and CAF1A KD on viral release. HeLa cells were infected with VSV-G pseudotyped HIV-Luc and treated with siRNAs at 24 hpi. A schematic of the experimental procedure is shown in (A). The expression of POLE3, POLE4, SMC5, and CAF1A was analyzed by immunoblotting at 72 hpi (B). Viral release in the supernatant was assessed by p24 antigen ELISA (C), and a luciferase assay (D) was performed at 72 hpi. The mean ± SD values of 4 independent experiments are plotted. The results showed that POLE3 KD did not affect viral release or expression from integrated HIV-1 DNA, similar to POLE4, SMC5 and CAF1A KD.

(E-F) Infectivity of HIV-1 virions produced in POLE3 KO HeLa cells. HeLa NT and POLE3 KO cells were infected with VSV-G pseudotyped NL4.3 (env+), and culture supernatant containing produced virions was collected at 48 hpi. The infectivity of virions produced in POLE3 KO and control cells was measured in TZM-bl cells. A schematic of the experimental procedure is shown in (E). HIV infectivity, corresponding to the ratio of luminescence to the quantity of p24 CA measured in the supernatant, is plotted (F). The results are presented as quantities relative to those in NT control cells, and the mean ± SD values of 2 independent experiments with triplicate samples are plotted.

* $P<0.05$, ** $P<0.01$, *** $P<0.001$; independent Student's t test.

Supplemental Table Legends

Table S1. HIV probes used for the PICh method. Thirty HIV probes were designed to cover the 5’LTR/Gag region of the HIV genome. The probes were composed of a desthiobiotin–TEG–2X spacer18 sequence followed by a 2’-fluoro-modified RNA/DNA oligonucleotide. Probes were synthetized by the Keck Foundation, Yale University.

Table S2. Factors identified by mass spectrometry in the eluates obtained in the PICh experiment performed in noninfected (NI) and infected (INF) SupT1 cells at 9 hpi. The results are presented as the number of peptides and the summed intensity of the peptides matched to the corresponding protein in the eluate and input fractions of INF and NI cells. Enrichment was determined as follows: #1. presence of the peptide in eluate-INF and absence in eluate-NI. #2. For peptides detected in both eluate-INF and eluate-NI, 2-fold more peptides were detected in eluate-INF than in eluate-NI. #3. The summed intensity was 3-fold higher in eluate-INF than in eluate-NI. Viral proteins are shown in green.

Table S3. Screening of pooled siRNAs for cell viability. HeLa cells were transfected with pooled siRNAs (455-siRNA library), and viability was assessed 48 hr later. siRNAs resulting in less than 75% viability were excluded (n=13). The results are presented as the mean of 2 independent experiments performed with triplicate samples.

Table S4. Screening of pooled siRNAs to identify factors involved in silencing of uHIV-1 DNA. HeLa cells were transfected with pooled siRNAs (455-siRNA library) and were infected with VSV-G pseudotyped HIV-Luc IN^{D116A} virus. Luciferase activity was measured 48 hr later. Forty-six siRNAs resulted in an increase of at least 1.93-fold in luciferase expression from uHIV-1 DNA. The results are presented as the mean of 2 independent experiments performed with triplicate samples.

Table S5. Screening of individual siRNAs for host factors regulating expression from uHIV-1 DNA. HeLa cells were transfected with the 4 individual siRNAs composing the pool used in the experiment referenced in Extended Data Tables 3-4. Cell viability was assessed 48 hr after transfection, and luciferase activity was measured 48 hpi with HIV-Luc IN^{D116A}. The results are presented as the mean of 3 (viability) or 6 (luciferase) independent experiments.

Table S6. **Validation of POLE3 and POLE4 KO clones by sequencing**. POLE3 and POLE4 KO HeLa clones and POLE3 KO HeLa-P4 clones generated using CRISPR-Cas9 gene editing were sequenced. DNA modifications upon gene KO and their effects on POLE3 and POLE4 protein expression are specified.

Table S7. Pooled siRNAs used in this study: siRNA library for the HIV-Luc IN^{D116A} screen and additional pooled siRNAs used for various experiments. Library of pooled siRNAs for the 455 hits identified by PICh. Gene, reference and gene ID are noted.

Table S8. Individual siRNAs used in this study: siRNA library for the HIV-Luc IND116A screen and additional siRNAs used for various experiments. The siRNA library was composed of 4 individual siRNAs targeting each of the 24 genes tested.

Table S9. Primers used in this study. List of primers used for HIV DNA quantification, ChIP, CUT&RUN, gene KD efficiency quantification and interferon response assessment.

Table S10. Antibodies used for WB, ChIP and CUT& RUN.

REFERENCES AND NOTES

1. R. D. Sloan, M. A. Wainberg, The role of unintegrated DNA in HIV infection. *Retrovirology* **8**, 52 (2011).

2. M. Lusic, R. F. Siliciano, Nuclear landscape of HIV-1 infection and integration. *Nat. Rev. Microbiol.* **15**, 69–82 (2017).

3. S. Machida, D. Depierre, H.-C. Chen, S. Thenin-Houssier, G. Petitjean, C. M. Doyen, M. Takaku, O. Cuvier, M. Benkirane, Exploring histone loading on HIV DNA reveals a dynamic nucleosome positioning between unintegrated and integrated viral genome. *Proc. Natl. Acad. Sci. U.S.A.* **117**, 6822–6830 (2020).

4. F. K. Geis, S. P. Goff, Unintegrated HIV-1 DNAs are loaded with core and linker histones and transcriptionally silenced. *Proc. Natl. Acad. Sci. U.S.A.* **116**, 23735–23742 (2019).

5. S. P. Goff, Silencing of Unintegrated Retroviral DNAs. *Viruses.* **13**, 2248 (2021).

6. J. Déjardin, R. E. Kingston, Purification of proteins associated with specific genomic Loci. *Cell* **136**, 175–186 (2009).

7. R. Bellelli, O. Belan, V. E. Pye, C. Clement, S. L. Maslen, J. M. Skehel, P. Cherepanov, G. Almouzni, S. J. Boulton, POLE3-POLE4 Is a Histone H3-H4 Chaperone that Maintains Chromatin Integrity during DNA Replication. *Mol. Cell* **72**, 112–126.e5 (2018).

8. R. A. Poot, G. Dellaire, B. B. Hülsmann, M. A. Grimaldi, D. F. Corona, P. B. Becker, W. A. Bickmore, P. D. Varga-Weisz, HuCHRAC, a human ISWI chromatin remodelling complex contains hACF1 and two novel histone-fold proteins. *EMBO J.* **19**, 3377–3387 (2000).

9. L. Dupont, S. Bloor, J. C. Williamson, S. M. Cuesta, R. Shah, A. Teixeira-Silva, A. Naamati, E. J. D. Greenwood, S. G. Sarafianos, N. J. Matheson, P. J. Lehner, The SMC5/6 complex compacts and silences unintegrated HIV-1 DNA and is antagonized by Vpr. *Cell Host Microbe* **29**, 792–805.e6 (2021).

10. F. K. Geis, Y. Sabo, X. Chen, Y. Li, C. Lu, S. P. Goff, CHAF1A/B mediate silencing of unintegrated HIV-1 DNAs early in infection. *Proc. Natl. Acad. Sci. U.S.A.* **119**, e2116735119 (2022).

11. S. L. Butler, M. S. Hansen, F. D. Bushman, A quantitative assay for HIV DNA integration in vivo. *Nat. Med.* **7**, 631–634 (2001).

12. P. Mohammadi, S. Desfarges, I. Bartha, B. Joos, N. Zangger, M. Muñoz, H. F. Günthard, N. Beerenwinkel, A. Telenti, A. Ciuffi, 24 hours in the life of HIV-1 in a T cell line. *PLOS Pathog.* **9**, e1003161 (2013).

13. C. M. Farnet, W. A. Haseltine, Determination of viral proteins present in the human immunodeficiency virus type 1 preintegration complex. *J. Virol.* **65**, 1910–1915 (1991).

14. M. D. Miller, C. M. Farnet, F. D. Bushman, Human immunodeficiency virus type 1 preintegration complexes: Studies of organization and composition. *J. Virol.* **71**, 5382–5390 (1997).

15. C. M. Farnet, F. D. Bushman, HIV-1 cDNA integration: Requirement of HMG I(Y) protein for function of preintegration complexes in vitro. *Cell* **88**, 483–492 (1997).

16. A. C. Francis, G. B. Melikyan, Single HIV-1 Imaging Reveals Progression of Infection through CA-Dependent Steps of Docking at the Nuclear Pore, Uncoating, and Nuclear Transport. *Cell Host Microbe* **23**, 536–548.e6 (2018).

17. M. Yamashita, A. N. Engelman, Capsid-Dependent Host Factors in HIV-1 Infection. *Trends Microbiol.* **25**, 741–755 (2017).

18. N. Hustedt, A. Álvarez-Quilón, A. McEwan, J. Y. Yuan, T. Cho, L. Koob, T. Hart, D. Durocher, A consensus set of genetic vulnerabilities to ATR inhibition. *Open Biol.* **9**, 190156 (2019).

19. B. Poon, I. S. Y. Chen, Human immunodeficiency virus type 1 (HIV-1) Vpr enhances expression from unintegrated HIV-1 DNA. *J. Virol.* **77**, 3962–3972 (2003).

20. B. Poon, M. A. Chang, I. S. Y. Chen, Vpr is required for efficient Nef expression from unintegrated human immunodeficiency virus type 1 DNA. *J. Virol.* **81**, 10515–10523 (2007).

21. G. Z. Wang, Y. Wang, S. P. Goff, Histones Are Rapidly Loaded onto Unintegrated Retroviral DNAs Soon after Nuclear Entry. *Cell Host Microbe* **20**, 798–809 (2016).

22. J. M. Kilzer, T. Stracker, B. Beitzel, K. Meek, M. Weitzman, F. D. Bushman, Roles of host cell factors in circularization of retroviral dna. *Virology* **314**, 460–467 (2003).

23. S. Munir, S. Thierry, F. Subra, E. Deprez, O. Delelis, Quantitative analysis of the time-course of viral DNA forms during the HIV-1 life cycle. *Retrovirology* **10**, 87 (2013).

24. N. Yan, A. D. Regalado-Magdos, B. Stiggelbout, M. A. Lee-Kirsch, J. Lieberman, The cytosolic exonuclease TREX1 inhibits the innate immune response to human immunodeficiency virus type 1. *Nat. Immunol.* **11**, 1005–1013 (2010).

25. C. Elsner, A. Ponnurangam, J. Kazmierski, T. Zillinger, J. Jansen, D. Todt, K. Döhner, S. Xu, A. Ducroux, N. Kriedemann, A. Malassa, P.-K. Larsen, G. Hartmann, W. Barchet, E. Steinmann, U. Kalinke, B. Sodeik, C. Goffinet, Absence of cGAS-mediated type I IFN responses in HIV-1-infected T cells. *Proc. Natl. Acad. Sci. U.S.A.* **117**, 19475–19486 (2020).

26. J. Rasaiyaah, C. P. Tan, A. J. Fletcher, A. J. Price, C. Blondeau, L. Hilditch, D. A. Jacques, D. L. Selwood, L. C. James, M. Noursadeghi, G. J. Towers, HIV-1 evades innate immune recognition through specific cofactor recruitment. *Nature* **503**, 402–405 (2013).

27. L. Zuliani-Alvarez, M. L. Govasli, J. Rasaiyaah, C. Monit, S. O. Perry, R. P. Sumner, S. McAlpine-Scott, C. Dickson, K. M. Rifat Faysal, L. Hilditch, R. J. Miles, F. Bibollet-Ruche, B. H. Hahn, T. Boecking, N. Pinotsis, L. C. James, D. A. Jacques, G. J. Towers, Evasion of cGAS and TRIM5 defines pandemic HIV. *Nat. Microbiol.* **7**, 1762–1776 (2022).

28. Y. Zhu, G. Z. Wang, O. Cingöz, S. P. Goff, NP220 mediates silencing of unintegrated retroviral DNA. *Nature* **564**, 278–282 (2018).

29. M. J. Pace, E. H. Graf, U. O'Doherty, HIV 2-long terminal repeat circular DNA is stable in primary CD4+T Cells. *Virology* **441**, 18–21 (2013).

30. B. Trinité, E. C. Ohlson, I. Voznesensky, S. P. Rana, C. N. Chan, S. Mahajan, J. Alster, S. A. Burke, D. Wodarz, D. N. Levy, An HIV-1 Replication Pathway Utilizing Reverse Transcription Products That Fail To Integrate. *J. Virol.* **87**, 12701–12720 (2013).

31. Y. Wu, J. W. Marsh, Selective transcription and modulation of resting T cell activity by preintegrated HIV DNA. *Science* **293**, 1503–1506 (2001).

32. B. Meltzer, D. Dabbagh, J. Guo, F. Kashanchi, M. Tyagi, Y. Wu, Tat controls transcriptional persistence of unintegrated HIV genome in primary human macrophages. *Virology* **518**, 241–252 (2018).

33. N. Kaplan, I. K. Moore, Y. Fondufe-Mittendorf, A. J. Gossett, D. Tillo, Y. Field, E. M. LeProust, T. R. Hughes, J. D. Lieb, J. Widom, E. Segal, The DNA-encoded nucleosome organization of a eukaryotic genome. *Nature* **458**, 362–366 (2009).

34. Y. Field, Y. Fondufe-Mittendorf, I. K. Moore, P. Mieczkowski, N. Kaplan, Y. Lubling, J. D. Lieb, J. Widom, E. Segal, Gene expression divergence in yeast is coupled to evolution of DNA-encoded nucleosome organization. *Nat. Genet.* **41**, 438–445 (2009).

35. E. Kenigsberg, A. Bar, E. Segal, A. Tanay, Widespread compensatory evolution conserves DNA-encoded nucleosome organization in yeast. *PLoS Comput. Biol.* **6**, e1001039 (2010).

36. Z. Zhang, C. J. Wippo, M. Wal, E. Ward, P. Korber, B. F. Pugh, A packing mechanism for nucleosome organization reconstituted across a eukaryotic genome. *Science* **332**, 977–980 (2011).

37. Y. Nakatani, V. Ogryzko, Immunoaffinity purification of mammalian protein complexes. *Methods Enzymol.* **370**, 430–444 (2003).

38. D. Kumar, J. L. Shadrach, A. J. Wagers, A. B. Lassar, Id3 is a direct transcriptional target of Pax7 in quiescent satellite cells. *Mol. Biol. Cell* **20**, 3170–3177 (2009).

39. B. Descours, G. Petitjean, J.-L. López-Zaragoza, T. Bruel, R. Raffel, C. Psomas, J. Reynes, C. Lacabaratz, Y. Levy, O. Schwartz, J. D. Lelievre, M. Benkirane, CD32a is a marker of a CD4 T-cell HIV reservoir harbouring replication-competent proviruses. *Nature* **543**, 564–567 (2017).

40. M. Ott, M. Schnölzer, J. Garnica, W. Fischle, S. Emiliani, H. R. Rackwitz, E. Verdin, Acetylation of the HIV-1 Tat protein by p300 is important for its transcriptional activity. *Curr. Biol.* **9**, 1489–1493 (1999).

41. I. du Chéné, E. Basyuk, Y.-L. Lin, R. Triboulet, A. Knezevich, C. Chable-Bessia, C. Mettling, V. Baillat, J. Reynes, P. Corbeau, E. Bertrand, A. Marcello, S. Emiliani, R. Kiernan, M. Benkirane, Suv39H1 and HP1gamma are responsible for chromatin-mediated HIV-1 transcriptional silencing and post-integration latency. *EMBO J.* **26**, 424–435 (2007).

42. S. L. Kan, N. Saksouk, J. Déjardin, Proteome Characterization of a Chromatin Locus Using the Proteomics of Isolated Chromatin Segments Approach. *Methods Mol. Biol.* **1550**, 19–33 (2017).

43. S. Ide, J. Dejardin, End-targeting proteomics of isolated chromatin segments of a mammalian ribosomal RNA gene promoter. *Nat. Commun.* **6**, 6674 (2015).

44. A. Brussel, P. Sonigo, Analysis of early human immunodeficiency virus type 1 DNA synthesis by use of a new sensitive assay for quantifying integrated provirus. *J. Virol.* **77**, 10119–10124 (2003).

45. U. O'Doherty, W. J. Swiggard, M. H. Malim, Human immunodeficiency virus type 1 spinoculation enhances infection through virus binding. *J. Virol.* **74**, 10074–10080 (2000).

46. C. F. Kessing, C. C. Nixon, C. Li, P. Tsai, H. Takata, G. Mousseau, P. T. Ho, J. B. Honeycutt, M. Fallahi, L. Trautmann, J. V. Garcia, S. T. Valente, In Vivo Suppression of HIV Rebound by Didehydro-Cortistatin A, a "Block-and-Lock" Strategy for HIV-1 Treatment. *Cell Rep.* **21**, 600–611 (2017).

47. G. Maarifi, J. Fernandez, D. M. Portilho, A. Boulay, J. Dutrieux, S. Oddos, G. Butler-Browne, S. Nisole, N. J. Arhel, RanBP2 regulates the anti-retroviral activity of TRIM5α by SUMOylation at a predicted phosphorylated SUMOylation motif. *Commun Biol.* **1**, 193 (2018).

48. X. Contreras, K. Salifou, G. Sanchez, M. Helsmoortel, E. Beyne, L. Bluy, S. Pelletier, E. Rousset, S. Rouquier, R. Kiernan, Nuclear RNA surveillance complexes silence HIV-1 transcription. *PLOS Pathog.* **14**, e1006950 (2018).

49. L. Rivière, B. Quioc-Salomon, G. Fallot, B. Halgand, C. Féray, M.-A. Buendia, C. Neuveut, Hepatitis B virus replicating in hepatocellular carcinoma encodes HBx variants with preserved ability to antagonize restriction by Smc5/6. *Antiviral Res.* **172**, 104618 (2019).

50. O. Molina, G. Vargiu, M. A. Abad, A. Zhiteneva, A. A. Jeyaprakash, H. Masumoto, N. Kouprina, V. Larionov, W. C. Earnshaw, Epigenetic engineering reveals a balance between histone modifications and transcription in kinetochore maintenance. *Nat. Commun.* **7**, 13334 (2016).

51. M. Ringeard, V. Marchand, E. Decroly, Y. Motorin, Y. Bennasser, FTSJ3 is an RNA 2'-O-methyltransferase recruited by HIV to avoid innate immune sensing. *Nature* **565**, 500–504 (2019).

52. B. Guey, M. Wischnewski, A. Decout, K. Makasheva, M. Kaynak, M. S. Sakar, B. Fierz, A. Ablasser, BAF restricts cGAS on nuclear DNA to prevent innate immune activation. *Science* **369**, 823–828 (2020).

53. D. Li, S. Swaminathan, Human IFIT proteins inhibit lytic replication of KSHV: A new feed-forward loop in the innate immune system. *PLOS Pathog.* **15**, e1007609 (2019).

54. M. Solis, P. Nakhaei, M. Jalalirad, J. Lacoste, R. Douville, M. Arguello, T. Zhao, M. Laughrea, M. A. Wainberg, J. Hiscott, RIG-I-mediated antiviral signaling is inhibited in HIV-1 infection by a protease-mediated sequestration of RIG-I. *J. Virol.* **85**, 1224–1236 (2011).

55. M. Morchikh, A. Cribier, R. Raffel, S. Amraoui, J. Cau, D. Severac, E. Dubois, O. Schwartz, Y. Bennasser, M. Benkirane, HEXIM1 and NEAT1 Long Non-coding RNA Form a Multi-subunit Complex that Regulates DNA-Mediated Innate Immune Response. *Mol. Cell* **67**, 387–399.e5 (2017).
